# Design Strategies of Cu-Based Tandem Catalysts for Efficient Electrochemical CO_2_ Reduction to C_2+_ Products

**DOI:** 10.1021/acs.nanolett.6c03203

**Published:** 2026-09-15

**Authors:** Yu-Cheng Liu, Yu-Jhih Shen, Kang-Shun Peng, Shuo-Peng Lin, Chi Kang, Chieh Chang Chien, Min-Si Lee, Shao-Hui Hsu, Sung-Fu Hung

**Affiliations:** † Department of Applied Chemistry and Center for Emergent Functional Matter Science, 34914 National Yang Ming Chiao Tung University, Hsinchu 300, Taiwan; ‡ Department of Medicinal and Applied Chemistry, Kaohsiung Medical University, Kaohsiung 807, Taiwan; § Taiwan Semiconductor Research Institute, 63353 National Institutes of Applied Research, Hsinchu 300, Taiwan

**Keywords:** CO_2_ electroreduction, flow cell, Cu-based catalysts, tandem catalysis

## Abstract

Electrochemical CO_2_ reduction reaction (CO_2_RR) offers a promising route to convert CO_2_ into value-added fuels and chemicals using renewable electricity. Cu-based catalysts are uniquely capable of producing C_2+_ hydrocarbons and oxygenates through C–C coupling, but their performance is limited by insufficient *CO coverage, broad product distributions, competing hydrogen evolution, and stability issues at high current densities. Tandem catalysis addresses these challenges by integrating CO-generating components with Cu-based C–C coupling sites. This review summarizes recent advances in Cu-based tandem catalysts under flow-cell and industrially relevant conditions, focusing on spatially separated systems, Ag–Cu bimetallic interfaces, single-atom alloys, and SAC–Cu tandem architectures. Key design principles include regulating CO generation, transport, interfacial structure, and coupling pathways.

## Graphical abstract

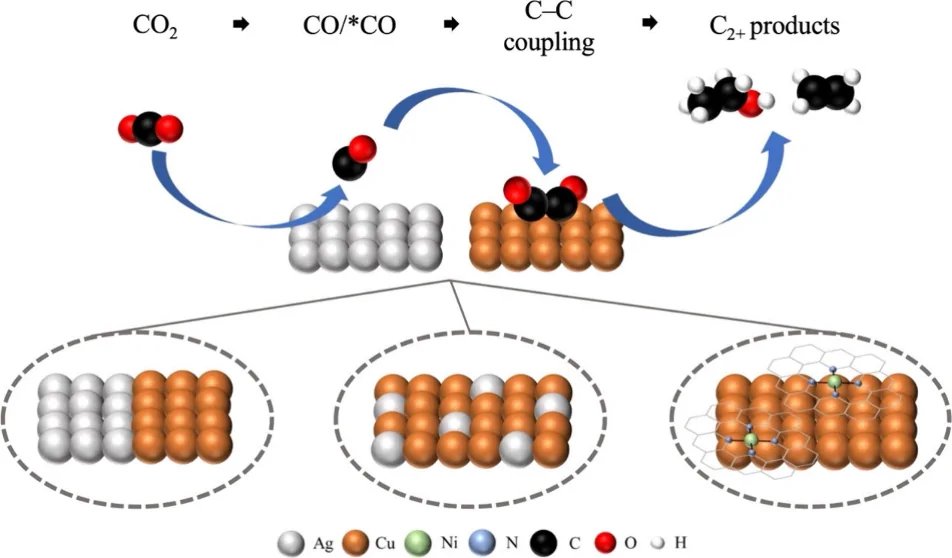

## 1 Introduction

Driven by the continuous rise in atmospheric CO_2_ concentrations and the growing climate impacts of greenhouse-gas emissions,^1–3^ converting CO_2_ from an emitted greenhouse gas into a reusable carbon feedstock has become a central objective of carbon capture, utilization, and storage (CCUS) technologies.^4–6^ Among the various CO_2_ conversion strategies, the electrochemical CO_2_ reduction reaction (CO_2_RR) has attracted considerable attention because it can be operated under relatively mild temperature and pressure conditions^7–9^ and can be directly powered by renewable electricity generated from solar, wind, and other sustainable energy sources.^10^ In contrast, photocatalytic CO_2_ conversion is often limited by insufficient light absorption, inefficient charge separation, and low quantum efficiency,^11,12^ whereas thermocatalytic CO_2_ conversion generally requires elevated temperature,^13^ high pressure,^14^ or external hydrogen sources.^15^ These advantages make electrocatalytic CO_2_RR a promising platform for coupling CO_2_ utilization with renewable-energy-driven chemical production.

Electrocatalytic CO_2_RR can produce a wide range of reduction products, including CO, formate, methane, methanol, ethylene, ethanol, and n-propanol.^16,17^ Among these products, C_1_ products such as CO and formate usually involve two-electron-transfer pathways and can therefore be produced with high selectivity over specific catalysts.^18,19^ In contrast, C_2+_ products, including ethylene, ethanol, and n-propanol, have attracted increasing interests owing to their higher energy density, greater economic value, and broad industrial relevance. Among the various CO_2_RR catalysts, Cu remains the most representative catalyst capable of producing C_2+_ products. Unlike Au, Ag, Sn, and Bi, which typically favor CO or formate formation,^20^ Cu exhibits moderate *CO adsorption strength, enabling CO intermediates to remain on the catalyst surface and participate in subsequent C–C coupling.^21^ Consequently, Cu is one of the few monometallic catalysts capable of electrochemically converting CO_2_ into hydrocarbons and oxygenates. However, because C_2+_ product formation involves multiple electron-transfer, proton-transfer, and C–C coupling steps, Cu-based CO_2_RR still faces major challenges, including broad product distributions, insufficient selectivity, and limited stability under high-current-density operation.^22–24^

To evaluate the industrial relevance of a CO_2_RR catalyst, several key performance metrics must be considered, including Faradaic efficiency (FE), partial current density, cell voltage, energy efficiency, and long-term stability. FE represents the fraction of electrons consumed to generate a target product and is therefore widely used to evaluate product selectivity. Partial current density, defined as the product of FE and total current density, reflects the actual production rate of a specific product. Techno-economic analyses (TEA) have suggested that economically viable CO_2_ electrolysis generally requires high current density, high FE, low cell voltage, and sufficient operational stability. Accordingly, an FE­(C_2+_) above approximately 70% and a C_2+_ partial current density on the order of 300 mA cm^–2^ are commonly regarded as important benchmarks for approaching industrially relevant CO_2_RR operation.^8,25,26^

However, conventional H-cell systems are generally unable to meet the requirements for high-current-density operation. Because CO_2_ has limited solubility in aqueous electrolytes and must first dissolve into the electrolyte before diffusing to the catalyst surface, CO_2_ mass transport in H-cells often becomes a major kinetic limitation. As a result, H-cell measurements are typically insufficient for accurately evaluating catalyst performance under practical operating conditions. To overcome CO_2_ transport limitations, recent CO_2_RR research has increasingly shifted toward gas-diffusion electrodes (GDEs) and flow-cell systems. These systems directly deliver gaseous CO_2_ to the vicinity of the catalyst layer and establish a gas–solid–liquid three-phase interface at the catalyst surface, thereby substantially shortening the CO_2_ transport distance and enabling CO_2_RR operation at current densities of several hundred mA cm^–2^ or even ampere-level current densities.^27–31^

The development of flow-cell CO_2_RR has brought Cu-based C_2+_ production closer to industrial implementation, but it has also introduced new challenges. Under high-current-density operation, insufficient *CO concentration and adsorption,^32^ electrolyte flooding,^33,34^ and competition from the hydrogen evolution reaction (HER) can significantly affect target-product formation. These challenges indicate that improving the intrinsic activity of Cu alone is insufficient for achieving efficient C_2+_ production. Instead, integrated catalyst design is required to regulate CO_2_/CO transport, surface intermediate coverage, local reaction environments, and C–C coupling pathways.^35^

In response to these challenges, tandem catalyst design has emerged as an important strategy for enhancing C_2+_ selectivity and production rates in Cu-based CO_2_RR. This approach generally involves integrating CO-generating components with Cu-based C–C coupling sites, enabling CO_2_ reduction, CO intermediate supply, and subsequent C–C coupling to proceed cooperatively within a single catalytic system.^36,37^ Therefore, this review focuses on recent design strategies and mechanistic insights into Cu-based tandem catalysts under high-current-density and flow-cell conditions, highlighting how different tandem architectures improve CO utilization and promote C_2+_ product formation.

## 2 Mechanisms and Tandem Design in Cu-Based CO_2_RR

Cu-based CO_2_RR toward C_2+_ products involves multiple proton-coupled electron transfer (PCET) steps and a series of complex surface-bound intermediates. As illustrated in Figure 1a, CO_2_ is generally first activated on the catalyst surface to form the *COOH intermediate, followed by further electron and proton transfer to generate *CO. For C_1_ products such as CO, *CO can directly desorb from the catalyst surface. In contrast, C_2+_ product formation requires sufficient *CO coverage on the Cu surface, allowing *CO intermediates to undergo subsequent C–C coupling.^38,39^ The most widely discussed pathway involves the dimerization of two *CO intermediates to form *OCCO, followed by protonation to generate key intermediates such as *OCCOH.^40,41^ Subsequent hydrogenation, protonation, and electron-transfer steps then lead to ethylene, ethanol, acetate, or other C_2_ products.^42–46^

**Figure 1:**
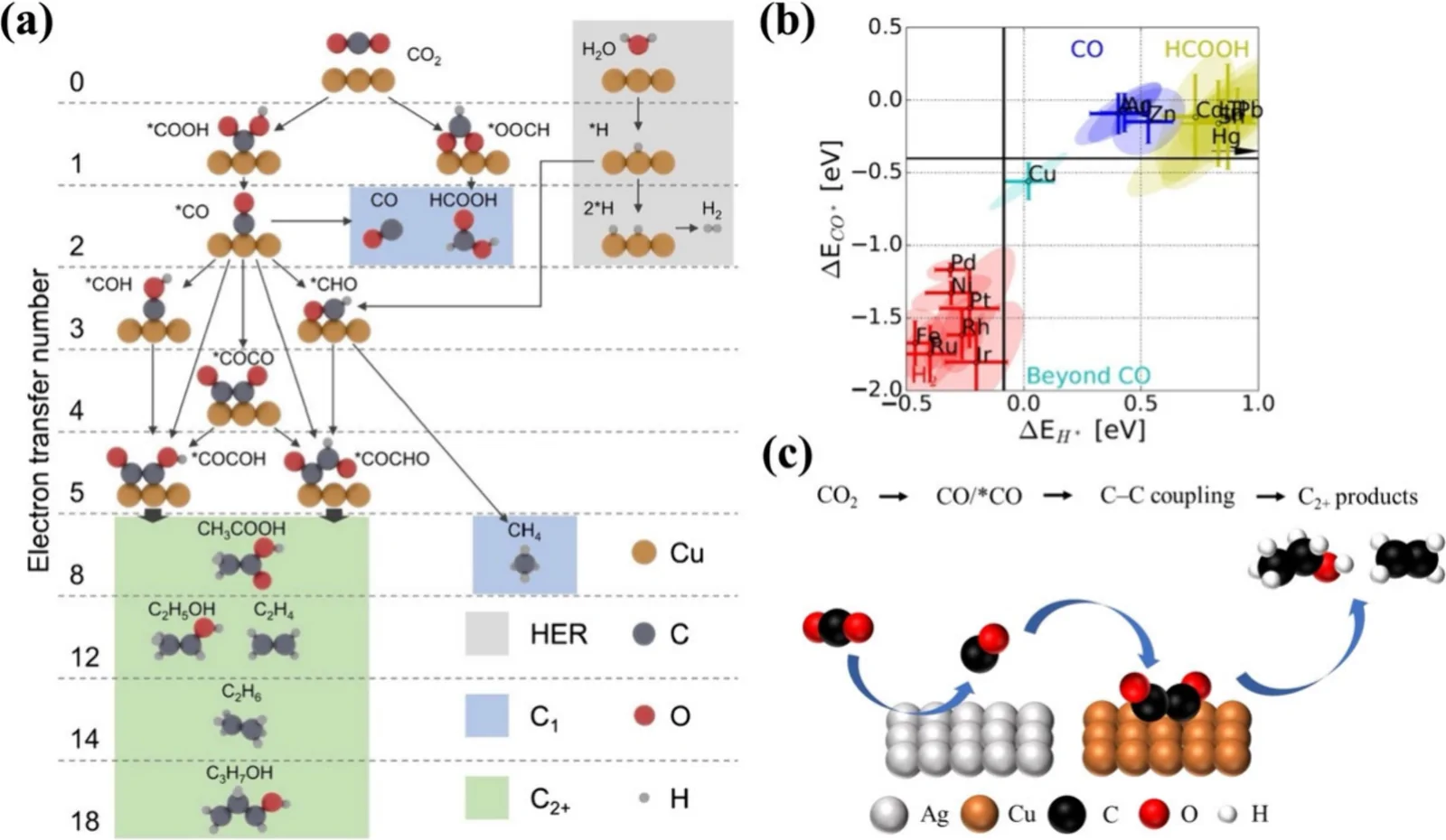
(a) Reaction pathways for CO_2_RR on Cu toward C_1_ and C_2+_ products. Reproduced from Ref 43. under the terms of the Creative Commons Attribution 4.0 International (CC BY 4.0) License. Copyright 2022 The Author(s). (b) Schematic relationship between intermediate adsorption strength and product selectivity on different metal catalysts. Reproduced with permission from Ref 21. Copyright 2017 Wiley-VCH GmbH & Co. KGaA. (c) General concept of Cu-based tandem catalysis, in which CO-generating components supply CO/*CO intermediates to adjacent Cu sites for C–C coupling and C_2+_ product formation.

In addition to the conventional *CO–*CO coupling pathway, recent studies have suggested that *CO may also couple with other C_1_ intermediates, such as *CHO^47^ or *CHx.^48^ The dominant reaction pathway depends strongly on the Cu crystal facet,^49,50^ local reaction microenvironment,^51–54^ surface *CO coverage,^32^ and applied potential.^55^ Therefore, although Cu is capable of producing various C_2+_ products, the complexity of these reaction pathways makes precise control over product selectivity highly challenging.^56,57^

The unique role of Cu in CO_2_RR primarily originates from its moderate adsorption strength toward key intermediates (Figure 1b).^21^ Compared with Au and Ag, which bind *CO weakly and therefore preferentially produce CO, and Sn or Bi, which tend to favor formate formation, Cu exhibits an intermediate *CO binding strength. This allows Cu to retain *CO intermediates on the surface without being completely poisoned by CO, thereby enabling further *CO hydrogenation and C–C coupling. However, this moderate adsorption strength also imposes an intrinsic limitation on Cu-based CO_2_RR. Under high-current-density operation, the *CO supply and surface *CO coverage required to sustain efficient C–C coupling may become insufficient. If *CO desorbs too rapidly, or if local CO_2_ depletion and competing HER reduce surface *CO coverage, the probability of C–C coupling decreases, resulting in lower C_2+_ selectivity and increased formation of C_1_ products or H_2_.^32,58–60^

As illustrated in Figure 1c, tandem catalysis provides a rational design strategy by assigning CO generation and C–C coupling to different but spatially adjacent catalytic sites. In a typical Cu-based tandem catalyst, a CO-generating component, such as Ag, Au, or M–N_4_ single-atom sites, preferentially reduces CO_2_ to CO or *CO intermediates. These CO intermediates then migrate to neighboring Cu sites through diffusion, spillover, or interfacial migration, where they undergo further C–C coupling and deep reduction to form C_2+_ products. The central objective of tandem catalysis is therefore to overcome insufficient *CO supply on Cu by increasing the local CO concentration and surface *CO coverage near Cu, while minimizing the transport distance between CO-generation sites and C–C coupling sites.

Another critical consideration in Cu-based tandem CO_2_RR is the competition with the hydrogen evolution reaction (HER), which directly influences both carbon utilization and C–C coupling. At the Cu surface, adsorbed *H intermediate generated during HER competes with *CO and other carbonaceous intermediates for available catalytic sites. Increased *H coverage can decrease the population and proximity of adsorbed *CO species required for carbon–carbon bond formation, thereby suppressing multicarbon production. Under commonly employed neutral or alkaline conditions, tandem architectures can mitigate HER through several complementary mechanisms. In particular, increasing the local supply and coverage of CO/*CO promotes preferential occupation of Cu surface sites by carbonaceous intermediates, thereby limiting competitive *H adsorption and maintaining the adjacent *CO configurations required for C–C coupling.^61,62^ In parallel, hydrophobic catalyst layers and gas-diffusion interfaces can regulate water accessibility and mitigate electrolyte flooding while preserving efficient CO_2_ and CO transport pathways.^63,64^ The combined regulation of surface-intermediate coverage and the local mass-transport environment therefore shifts the competition from *H formation toward carbonaceous intermediate retention and C–C coupling.

The competition between CO_2_RR and HER becomes considerably more complex under acidic conditions, where the high proton availability intrinsically favors hydrogen evolution. Although acidic CO_2_ electrolysis offers important advantages by mitigating carbonate formation and improving carbon utilization, the tandem enhancement commonly observed for Ag–Cu systems under neutral or alkaline conditions can be substantially weakened. Beyond increased competition from *H adsorption, the acidic electrolyte can directly modify the adsorption behavior and interfacial state of Cu. Experimental and theoretical studies have shown that hydronium ions can weaken *CO binding on Cu,^65^ thereby reducing the retention and utilization efficiency of tandem- generated CO. Strongly acidic environments can also deplete surface oxygen-containing Cu surface species that have been associated with favorable C–C coupling.^66^ These effects indicate that proton activity influences tandem catalysis not only through direct competition for surface sites but also by altering the chemical state and adsorption properties of the Cu interface.

Interestingly, recent studies of acidic tandem CO_2_RR demonstrate that the CO-generating component can perform an additional function beyond simply supplying CO. By consuming protons and modifying the local reaction environment, the secondary catalytic component can establish a neutral-to-alkaline microenvironment around Cu, thereby suppressing proton-driven HER and restoring favorable oxygen-containing surface species favorable for C–C coupling.^66,67^ Furthermore, the composition and spatial distribution of Ag within CuAg tandem catalysts can reorganize the interfacial water network and consequently regulate the balance between *CO retention and *H adsorption.^68^ These findings demonstrate that HER suppression in tandem CO_2_RR should not be regarded simply as minimizing H_2_ production. Rather, it involves coordinated control over *CO/*H competitive adsorption, local CO coverage, proton and water accessibility, local pH, Cu surface chemistry, and the interfacial solvation environment. Rational regulation of these coupled factors is therefore essential for maintaining efficient intermediate utilization and C–C coupling across different electrolyte environments.

However, recent studies have increasingly shown that efficient tandem catalysis cannot be achieved solely by increasing CO generation. If the generated CO desorbs, escapes from the catalyst layer, or diffuses into the electrolyte before participating in C–C coupling, the overall CO utilization efficiency remains limited. Therefore, in addition to enhancing CO/*CO availability, precise control over CO transport, retention, adsorption, intermediate migration, and coupling pathways on Cu surfaces has become a central consideration in the design of Cu-based tandem catalysts.

In recent years, advances in flow-cell and gas-diffusion-electrode technologies have enabled Cu-based tandem catalysts to achieve substantially improved C_2+_ selectivity and partial current density under commercially relevant high-current-density conditions, highlighting their potential for practical CO_2_RR applications. As summarized schematically in Figure 2, the tandem catalysts discussed in this review can be categorized into four representative architectures according to the spatial relationship and degree of structural integration between the CO-generating sites and Cu: spatially separated tandem catalysts, bimetallic heterointerfaces, single-atom alloys (SAAs), and SAC–Cu tandem systems. Although all four architectures share the fundamentally objective of increasing the local availability and effective utilization of CO/*CO intermediates for subsequent C–C coupling on Cu, they accomplish this through distinct intermediate-transfer pathways and interfacial regulatory mechanisms.

**Figure 2:**
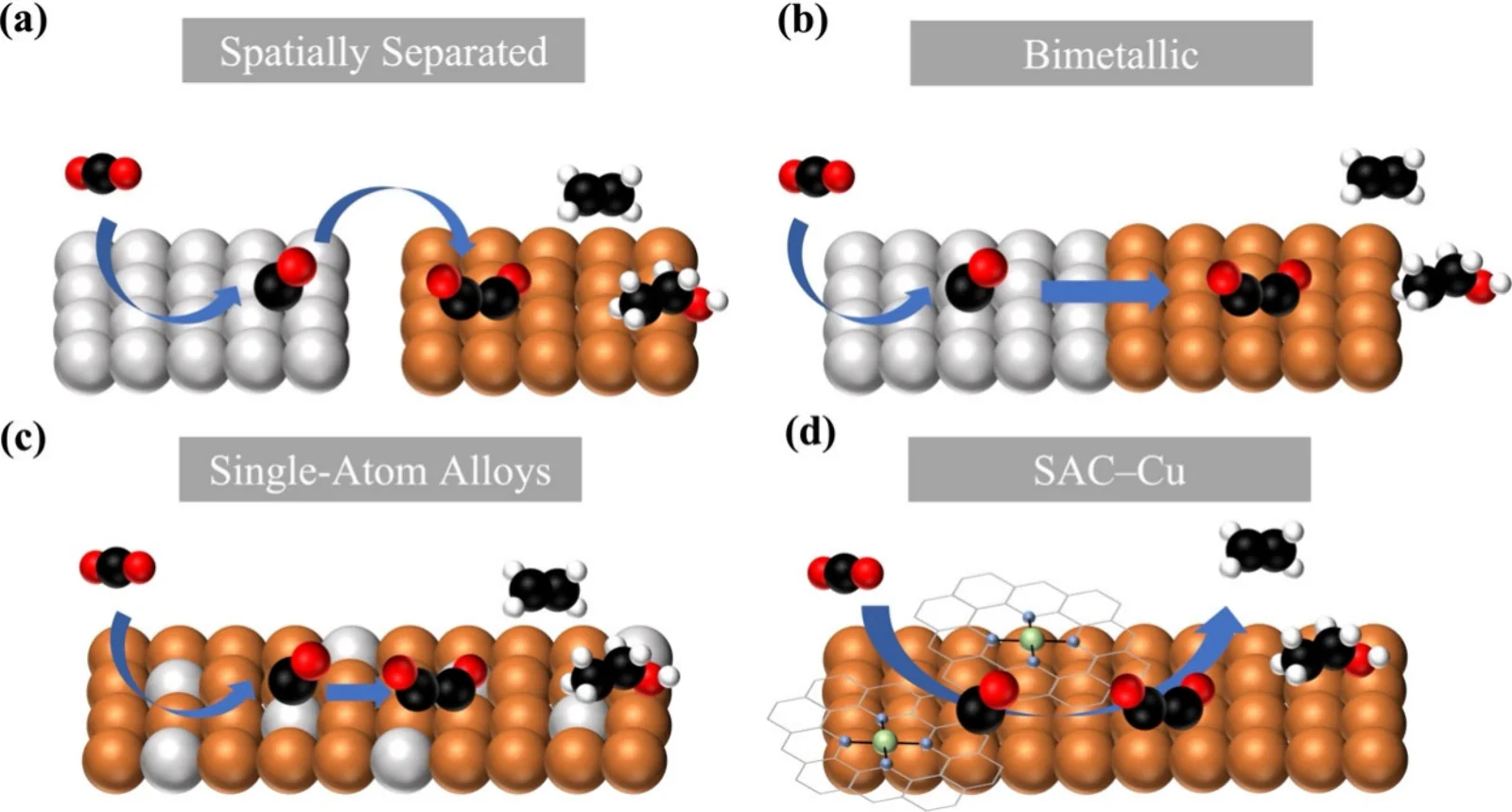
Schematic comparison of four representative Cu-based tandem catalyst architectures and their regulatory mechanisms for CO_2_ electroreduction toward C_2+_ products. (a) In spatially separated tandem systems, CO generated on a physically distinct catalytic component is transported to Cu through diffusion or spillover, with tandem efficiency strongly affected by CO residence time, transport distance, and retention. (b) Bimetallic heterointerfaces establish direct contact between the CO-generating component and Cu, enabling short-range intermediate transfer together with interfacial electronic modulation and regulation of intermediate adsorption. (c) Single-atom alloys incorporate isolated secondary-metal atoms into the Cu lattice, allowing atomic-scale intermediate inter-migration and local electronic modulation while preserving contiguous Cu ensembles for C–C coupling. (d) SAC–Cu tandem systems employ isolated metal coordination sites as tunable CO-generating centers, followed by CO/*CO transfer to neighboring Cu sites, with potential alignment and coordination-site stability playing important roles. After transfer to Cu, the supplied CO/*CO intermediates undergo C–C coupling through *CO–*CO or asymmetric coupling pathways to form C_2+_ products. The principal limitations associated with each tandem architecture are also indicated.

In spatially separated systems, CO is generated on a physically distinct catalytic component and subsequently transported to Cu through diffusion or spillover Consequently, the transport distance, residence time, and retention of CO within the catalyst layer strongly influence the probability that the generated intermediate reaches Cu before escaping into the gas phase. Bimetallic tandem catalysts reduce this spatial separation by establishing direct phase boundaries between the CO-generating component and Cu/CuO_x_. In addition to shortening the intermediate-transfer distance, these heterointerfaces can induce charge redistribution, stabilization of favorable Cu oxidation states, and modify intermediate adsorption energetics, thereby coupling CO supply with interfacial regulation of C–C coupling.

SAAs provide a higher degree of structural integration by incorporating isolated secondary-metal atoms directly into the Cu lattice. This atomic-scale arrangement enables local electronic and geometric modulation of neighboring Cu sites while maintaining contiguous Cu ensembles required for C–C bond formation. The close proximity between the isolated atoms and Cu further facilitates short-range inter-migration of CO/*CO intermediates, minimizing the transport distance between complementary catalytic sites. In SAC–Cu tandem systems, by contrast, atomically dispersed coordination sites, such as M–N_4_ centers or molecular complexes, serve as independently tunable CO-generating centers coupled to Cu. Their tandem performance therefore depends not only on intrinsic CO-generation activity but also on potential-window matching between the two catalytic components, effective interfacial contact, and efficient delivery of the generated CO/*CO intermediates to Cu.

Following transfer to Cu, the local enriched CO/*CO intermediates can undergo conventional *CO–*CO coupling or asymmetric coupling with hydrogenated C_1_ intermediates, ultimately promoting C_2+_ product formation. Thus, these four tandem architectures regulate a common reaction sequence at different structural length scales, ranging from mesoscale CO transport in spatially separated systems and phase-boundary-mediated interactions in bimetallic catalysts to atomic-scale intermediate migration in SAAs and coordination-site engineering in SAC-Cu systems. Their major limitations consequently differ: spacially separated system are particularly susceptible to CO loss and mass-transport limitations; bimetallic catalysts may undergo dynamic interfacial reconstruction; SAAs require simultaneous stabilization of isolated atoms and preservation of contiguous Cu ensembles; and SAC-Cu systems depend strongly on coordination-site stability, potential matching, and efficient coupling between the CO-generating sites and Cu. This architecture-dependent perspective provides a framework for understanding how the spatial organization of complementary catalytic sites governs intermediate transfer and ultimately determines tandem CO_2_RR performance.

## 3 Spatially Separated Tandem Catalysts

In CO_2_RR tandem catalytic systems, coupling Cu with CO-producing metal catalysts, such as Ag or Au, has been widely demonstrated as an effective strategy for enhancing C_2+_ product selectivity. This improvement is generally attributed to the increased local concentration of the key *CO intermediate near Cu surfaces, which promotes the kinetics of C–C coupling. In an early study, Chen et al. showed that the physical mixing of commercial Cu and Ag powders could significantly improve C_2+_ production in CO_2_RR. In 1 M KOH at −0.70 V versus RHE, the incorporation of Ag increased the partial current density of C_2+_ products from 37 to 160 mA cm^–2^,^59^ indicating that Ag-derived CO can effectively serve as a precursor intermediate for subsequent C–C coupling on Cu.

However, these early tandem strategies also revealed an important limitation: simply increasing CO supply near Cu does not necessarily lead to highly efficient C_2+_ formation. Because the *CO binding strength on Cu is relatively limited, a substantial fraction of the generated CO may desorb or escape from the catalyst layer before participating in C–C coupling, thereby decreasing CO utilization efficiency and limiting C–C coupling performance. Consequently, recent designs of spatially separated tandem catalysts have gradually shifted from merely increasing local CO concentration toward more comprehensive CO management strategies, including CO generation, transport, residence, adsorption, trapping, and selective conversion on Cu surfaces.

### 3.1 Nanowire Arrays and Hydrophobic Microenvironments for Promoting CO Transport, Retention, and Utilization

Recent studies have demonstrated that nanostructured arrays can improve CO utilization efficiency within the catalyst layer by prolonging CO residence time and increasing the probability of C–C coupling.^69–73^ Bian et al. designed a hierarchical Cu NWs/NC@Ag-PTFE tandem electrode to enhance the spillover, adsorption, and C–C coupling of Ag-derived CO.^74^ As shown in Figure 3a, this electrode consists of a PTFE membrane, an Ag layer, vertically aligned Cu nanowire arrays, and a nitrogen-doped carbon (NC) modification layer. The Ag layer functions as the CO-generating component, preferentially reducing CO_2_ to CO, whereas the inner Cu nanowire arrays receive Ag-derived CO through spillover. The vertically aligned and porous Cu nanowire arrays increase the CO diffusion path and residence time within the catalyst layer, thereby enhancing the probability that CO contacts Cu reaction sites and participates in subsequent coupling reactions.

**Figure 3:**
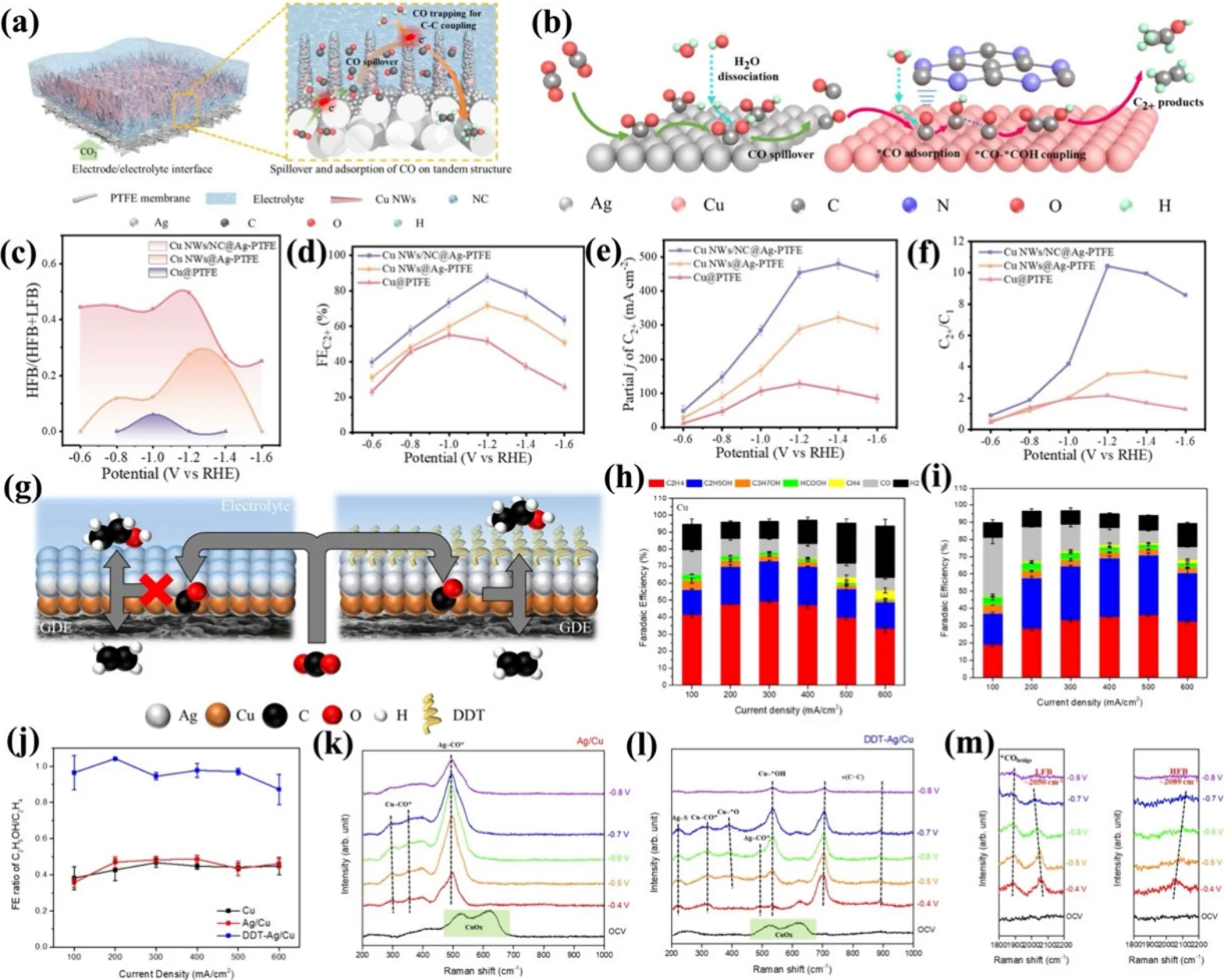
(a) Schematic illustration of the Cu NWs/NC@Ag-PTFE tandem electrode structure and the electrode/electrolyte interface. (b) Schematic illustration of CO spillover, CO adsorption, H_2_O dissociation, and *CO–*CO coupling on the Cu NWs/NC@Ag-PTFE tandem structure. (c) Potential-dependent HFB/(HFB + LFB) ratios obtained from in situ Raman analysis of *CO adsorption on Cu NWs/NC@Ag-PTFE, Cu NWs@Ag-PTFE, and Cu@PTFE. (d) Faradaic efficiency of C_2+_ products on Cu NWs/NC@Ag-PTFE, Cu NWs@Ag-PTFE, and Cu@PTFE at different applied potentials. (e) Partial current density of C_2+_ products on Cu NWs/NC@Ag-PTFE, Cu NWs@Ag-PTFE, and Cu@PTFE at different applied potentials. (f) C_2+_/C_1_ product ratio on Cu NWs/NC@Ag-PTFE, Cu NWs@Ag-PTFE, and Cu@PTFE at different applied potentials. Panels (a–f) reproduced with permission from Ref 74. Copyright 2025 American Chemical Society. (g) Schematic illustration of electrolyte flooding in unmodified Ag/Cu and hydrophobic regulation in DDT–Ag/Cu electrodes. (h, i) Product Faradaic efficiency distributions on Ag/Cu and DDT–Ag/Cu electrodes under different current densities. (j) Ethanol-to-ethylene Faradaic efficiency ratio on Cu, Ag/Cu, and DDT–Ag/Cu electrodes at different current densities. (k, l) In situ Raman spectra of Ag/Cu and DDT–Ag/Cu electrodes collected at different applied potentials. (m) In situ Raman spectra in the CO stretching region showing LFB and HFB features under different applied potentials. Panels (g–m) reproduced from Ref 64. under the terms of the Creative Commons Attribution 4.0 International (CC BY 4.0) License. Copyright 2026 The Author(s).

Beyond the physical transport advantages provided by the nanowire arrays, the NC modification layer further contributes to CO trapping and intermediate stabilization.^75^ Compared with Cu NWs@Ag-PTFE without NC modification, the Cu/NC interface in Cu NWs/NC@Ag-PTFE strengthens *CO adsorption, enabling Ag-derived CO to be more effectively confined near Cu reaction sites. In situ spectroscopic analysis revealed that this hierarchical structure enhances the signal of linear *CO intermediates, promotes H_2_O dissociation, and stabilizes the key C–C coupling intermediate *OCCOH, as schematically illustrated in Figure 3b. In situ Raman analysis of *CO adsorption further showed that Cu NWs/NC@Ag-PTFE exhibits a higher high-frequency band (HFB) ratio relative to the low-frequency band (LFB), suggesting higher *CO coverage or a more favorable adsorption environment for adjacent *CO species on the Cu surface, as shown in Figure 3c.^76,77^

In terms of catalytic performance, Cu NWs/NC@Ag-PTFE achieved a C_2+_ Faradaic efficiency of 87.5% at a current density of 519.0 mA cm^–2^ and exhibited a high C_2+_/C_1_ ratio of 10.42, significantly outperforming Cu NWs@Ag-PTFE and Cu@PTFE (Figure 3d–f). These results indicate that the hierarchical architecture not only increases CO supply but also improves CO utilization efficiency on Cu. In particular, the enhanced C_2+_/C_1_ ratio suggests that a greater fraction of CO intermediates is directed toward C–C coupling rather than being released from the reaction system as CO or converted into other C_1_ products.

In addition to CO transport and trapping within the catalyst layer, interfacial wettability under flow-cell conditions is another key factor determining whether the tandem effect can be fully activated.^78^ Because CO_2_RR occurs at a gas–solid–liquid three-phase interface, excessive hydrophilicity of the Ag/Cu electrode surface or electrolyte flooding of gas-diffusion pathways can restrict CO_2_ access to Ag sites, thereby suppressing Ag-mediated CO generation. Even when Ag and Cu are spatially adjacent, insufficient CO_2_ supply or disruption of the CO transfer pathway can prevent the intrinsic Ag–Cu tandem effect from being effectively expressed.

Interfacial wettability is a critical design parameter in tandem CO_2_ electroreduction because it governs the balance among CO_2_ transport, water accessibility, electrolyte penetration, and intermediate utilization. Importantly, wettability can be regulated across multiple length scales, ranging from the catalyst surface and ionomer/binder environment to gas-diffusion layer (GDL). For example, Lee et al.^63^ employed Cu/Ag bilayer electrodes incorporating cation- and anion-conducting ionomers and demonstrated that optimization of the ionomer configuration and local hydrophobicity could simultaneously regulate the interfacial pH and water content. This tailored microenvironment suppressed the competing HER while facilitating the conversion of tandem-generated CO toward C_2+_ products, highlighting the important role of ionomers in controlling both mass transport and the local reaction environment.

Beyond catalyst-layer engineering, Li et al.^79^ developed a hydrophobicity-graded gas-diffusion layer for Ni single-atom/Cu tandem electrodes. The spatially tailored hydrophobicity mitigated electrolyte flooding and maintained efficient CO_2_ transport, while in situ Raman spectroscopy revealed increased local CO intermediate coverage. These effects promoted ethylene formation and substantially improved operational stability, demonstrating that GDL-level wettability engineering can directly regulate intermediate availability and utilization. More recently, Park et al.^80^ systematically investigated the binder wettability in layered Ag/Cu tandem electrodes and identified an optimal configuration consisted of a relatively hydrophilic CO-producing layer coupled with a hydrophobic Cu layer. These findings demonstrate that maximizing the overall hydrophobicity of tandem electrode is not necessarily beneficial. Instead, spatially differentiated wettability should be rationally tailored across the functional layers to balance CO_2_ transport, water accessibility, electrolyte management, HER suppression, and CO intermediate utilization, thereby promoting selective and stable C_2+_ production.

Liu et al. investigated the effect of interfacial wettability on layered Ag/Cu electrodes in a flow cell.^64^ The results showed that the Ag/Cu electrode exhibited CO_2_RR performance similar to that of the Cu-only electrode, indicating that the Ag/Cu tandem effect was suppressed under practical operating conditions. This suppression was mainly attributed to the exposed Ag layer, which rendered the electrode surface more susceptible to electrolyte wetting, thereby hindering CO_2_ transport to Ag sites and decreasing both CO generation and subsequent CO transfer.

To address this issue, they employed a 1-dodecanethiol (DDT) self-assembly strategy to form a molecular hydrophobic layer on the Ag/Cu surface through Ag–S bonding. After DDT modification, the electrode surface became significantly more hydrophobic, effectively reducing electrolyte intrusion and maintaining a gas-accessible Ag/Cu interface. This ultrathin hydrophobic layer enabled more efficient CO_2_ transport to Ag sites and facilitated the transfer of Ag-generated CO to the Cu surface. Because the DDT layer is extremely thin, it improves surface hydrophobicity while avoiding excessive reactant blocking or significant mass-transfer resistance, as illustrated in Figure 3g.

Catalytically, DDT–Ag/Cu exhibited markedly improved C_2+_ production compared with unmodified Ag/Cu and Cu electrodes, as reflected in the product distributions shown in Figure 3h, i. Figure 3j further shows that DDT modification increased the ethanol-to-ethylene ratio from approximately 0.5 to approximately 1.0. This result suggests that hydrophobic surface modification not only restores the Ag/Cu tandem effect but may also affect the C–C coupling pathway on Cu by regulating local CO_2_/CO availability, CO residence time, and the reaction microenvironment. The in situ Raman results shown in Figure 3k–m reveal the appearance of Cu–*OH after hydrophobic modification. In addition, the HFB signal is relatively stronger on DDT–Ag/Cu, indicating that the Cu surface may possess a higher-coverage *CO adsorption environment after hydrophobic modification. Since HFB is generally associated with a high-*CO-coverage state favorable for C–C coupling, these results further suggest that the DDT hydrophobic layer not only improves gas transport but also promotes C_2+_ formation by maintaining high local CO availability.

Taken together, these studies indicate that the central challenge in spatially separated Ag/Cu tandem catalysts is not merely to increase CO generation, but to ensure that CO can be effectively transported, retained, and directed toward C–C coupling on Cu under flow-cell conditions.

### 3.2 Cu Facet Regulation and Asymmetric C–C Coupling for Controlling Product Distribution

Another important design direction for tandem catalysts is the precise regulation of the reaction pathway of tandem-generated CO on Cu surfaces. Traditional Ag/Cu tandem catalysts primarily aim to increase the local CO concentration near Cu, thereby promoting *CO–*CO coupling and C_2_^+^ product formation. However, recent studies have shown that, when specific hydrogenated carbon intermediates are stabilized on the Cu surface, Ag-derived CO does not necessarily participate exclusively in the conventional *CO–*CO coupling pathway. Instead, it can undergo asymmetric C–C coupling with *CHx species on Cu, thereby altering the C_2_ product distribution and, in particular, enhancing ethanol selectivity.^81,82^

Luan et al. designed a layered Cu/Ag tandem catalyst composed of Cu(111)-oriented nanosheets and Ag nanoparticles to redirect CO_2_RR from a hydrocarbon-dominant pathway toward oxygenate production, as shown in Figure 4a.^48^ On bare Cu(111), the reaction mainly proceeds through successive hydrogenation, where carbon intermediates generated from CO_2_ reduction are progressively hydrogenated into *CHx species and further converted into hydrocarbons.^83^ In contrast, in the layered Cu/Ag tandem catalyst, Ag nanoparticles act as CO-generating sites, reducing CO_2_ to CO and enabling CO intermediates to diffuse to the adjacent Cu(111) surface. Meanwhile, the Cu(111) facet favors the formation of hydrogenated carbon intermediates, such as *CH_2_ or *CH. When Ag-derived CO and Cu(111)-derived *CHx species coexist, the reaction can proceed through asymmetric *CH_2_–CO or *CH–CO coupling pathways to form oxygenated C_2_ intermediates, thereby promoting ethanol formation.

**Figure 4:**
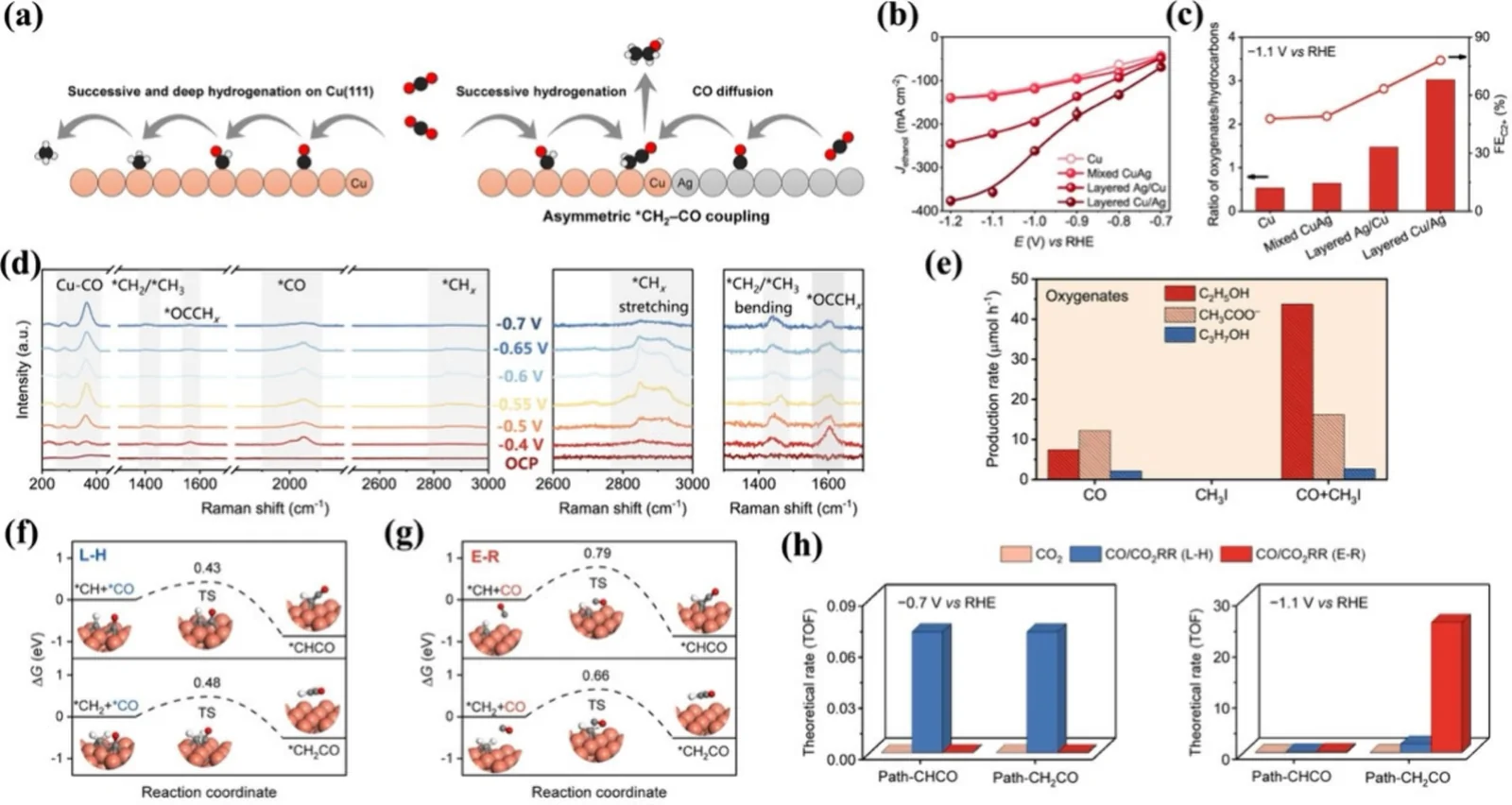
(a) Schematic illustration comparing successive hydrogenation on Cu(111) and asymmetric *CH_2_–CO coupling on layered Cu/Ag tandem catalysts. (b) Ethanol partial current density of Cu, mixed CuAg, layered Ag/Cu, and layered Cu/Ag catalysts at different applied potentials. (c) Oxygenates-to-hydrocarbons ratio and FE­(C_2+_) of Cu, mixed CuAg, layered Ag/Cu, and layered Cu/Ag catalysts at −1.1 V vs. RHE. (d) Operando Raman spectra of layered Cu/Ag collected at different applied potentials, showing Cu–CO, *CO, *CHx, *CH_2_/*CH_3_, and *OCCHx-related vibrational features. (e) Production rates of oxygenated products under CO, CH_3_I, and CO + CH_3_I co-feeding conditions. (f) DFT-calculated reaction energy profiles for Langmuir–Hinshelwood-type asymmetric coupling pathways involving *CH + CO and *CH_2_ + CO. (g) DFT-calculated reaction energy profiles for Eley–Rideal-type asymmetric coupling pathways involving *CH + CO and *CH_2_ + CO. (h) Theoretical rates of *CHCO and *CH_2_CO formation pathways under CO_2_, CO/CO_2_RR-LH, and CO/CO_2_RR-ER conditions. Reproduced with permission from Ref 48. Copyright 2024 American Chemistry Society.

The electrochemical tests and product analysis shown in Figure 4b, c demonstrate that the reaction pathway on layered Cu/Ag indeed shifts from hydrocarbon-dominant to oxygenate-enhanced. Compared with bare Cu, mixed CuAg, and Ag/Cu structures, layered Cu/Ag exhibits a higher ethanol partial current density over a wide potential range. In particular, at −1.1 V versus RHE, the oxygenates-to-hydrocarbons ratio of layered Cu/Ag is much higher than those of the control catalysts, while the FE­(C_2+_) is also significantly enhanced. These results indicate that the advantage of layered Cu/Ag lies not only in increasing overall C_2+_ formation, but also in steering the C_2+_ product distribution toward oxygenated products such as ethanol.

Operando Raman spectroscopy, shown in Figure 4d, provides important evidence for this altered reaction pathway. On the layered Cu/Ag catalyst, as the applied potential shifts negatively, signals associated with Cu–CO, *CO, *CHx stretching, *CH_2_/*CH_3_ bending, and *OCCHx species can be observed.^84–88^ This indicates that CO intermediates and hydrogenated carbon species are simultaneously formed on the Cu surface. These spectroscopic features support the occurrence of CO diffusion and *CHx-assisted asymmetric coupling in layered Cu/Ag. Compared with the successive hydrogenation pathway dominant on pure Cu(111), layered Cu/Ag enables a bifunctional synergy in which Ag supplies CO while Cu(111) generates *CHx species, allowing cross-coupling between *CHx and CO and thereby modifying C_2_ product selectivity.

The co-feeding experiment shown in Figure 4e further verifies the importance of *CHx–CO asymmetric coupling for ethanol formation.^81^ When only CO was introduced, the oxygenate production rate remained relatively limited. In contrast, when CO and CH_3_I were co-fed, the oxygenate production rate increased significantly, especially for ethanol. This result demonstrates that increasing CO availability while simultaneously providing a source of hydrogenated carbon intermediates can facilitate the asymmetric C–C coupling pathway. In other words, the coexistence of Ag-derived CO and Cu(111)-derived *CHx species is critical for achieving ethanol-selective CO_2_RR on layered Cu/Ag.

The DFT calculations shown in Figure 4f–h compare Langmuir–Hinshelwood (L–H) and Eley–Rideal-type asymmetric coupling routes. The results indicate that, in layered Cu/Ag, Ag-derived CO can couple with Cu(111)-derived *CH or *CH_2_ species to form *CHCO or *CH_2_CO intermediates. In particular, under more negative potentials, the *CH_2_ + CO coupling pathway exhibits a higher theoretical rate, supporting the conclusion that CO diffusion and *CHx-assisted asymmetric coupling are key factors in improving ethanol selectivity.

Overall, this section demonstrates that spatially separated tandem catalysts have evolved beyond the simple enhancement of local CO concentration. To further improve C_2+_ selectivity and production rates under industrially relevant CO_2_RR conditions, spatial tandem catalyst design should integrate CO management with precise control over the C–C coupling pathway.

## 4 Ag–Cu Bimetallic and Single-Atom Alloys

In conventional spatially separated Ag/Cu tandem catalysts, CO generated on Ag sites must diffuse or spill over to Cu surfaces before participating in C–C coupling. Consequently, the overall tandem efficiency can be strongly influenced by interfacial distance, CO residence time, and mass transport. By contrast, Ag–Cu bimetallic interfaces and AgCu single-atom alloys enable closer integration of CO generation, *CO migration, and C–C coupling within intimate metal–metal or metal–oxide interfaces environments. These systems can further promote C_2+_ formation through electronic-structure modulation, stabilization of Cu oxidation states, and atomic-level inter-migration of reaction intermediates.

### 4.1 Ag–Cu Bimetallic Catalysts: Interfacial and Electronic-Structure Modulation for Promoting C–C Coupling

In Ag–Cu bimetallic systems, the role of Ag extends beyond that of a simple CO supplier. Ag incorporation can increase the *CO coverage near adjacent Cu sites, modify the electronic state of Cu, and regulate the energetic barriers associated with CO_2_ activation, *CO formation, and *CO dimerization. As a result, Ag–Cu bimetallic interfaces provide a more tightly coupled cooperative reaction mode than conventional spatially separated tandem catalysts.

Wei et al. reported an Ag/Cu_2_O interfacial catalyst for highly selective ethylene production under neutral CO_2_RR conditions.^89^ In this system, Ag was introduced onto the Cu_2_O surface to form a phase-separated but intimately contacted Ag/Cu_2_O heterointerface, rather than a simple physical mixture, as shown in Figure 5a. Electronic-structure analysis by XPS showed that the Cu 2p and Ag 3d signals of the Ag–Cu_2_O catalyst shifted noticeably compared with those of pristine Cu_2_O and Ag, indicating the formation of electron-deficient Cu species and strong electronic interactions among Ag, Cu, and O atoms in Cu_2_O (Figure 5b,c). This interfacial electronic modulation alters the electronic state of Cu sites at the Ag/Cu_2_O interface, thereby increasing *CO coverage around Cu active sites and promoting subsequent C–C coupling.

**Figure 5:**
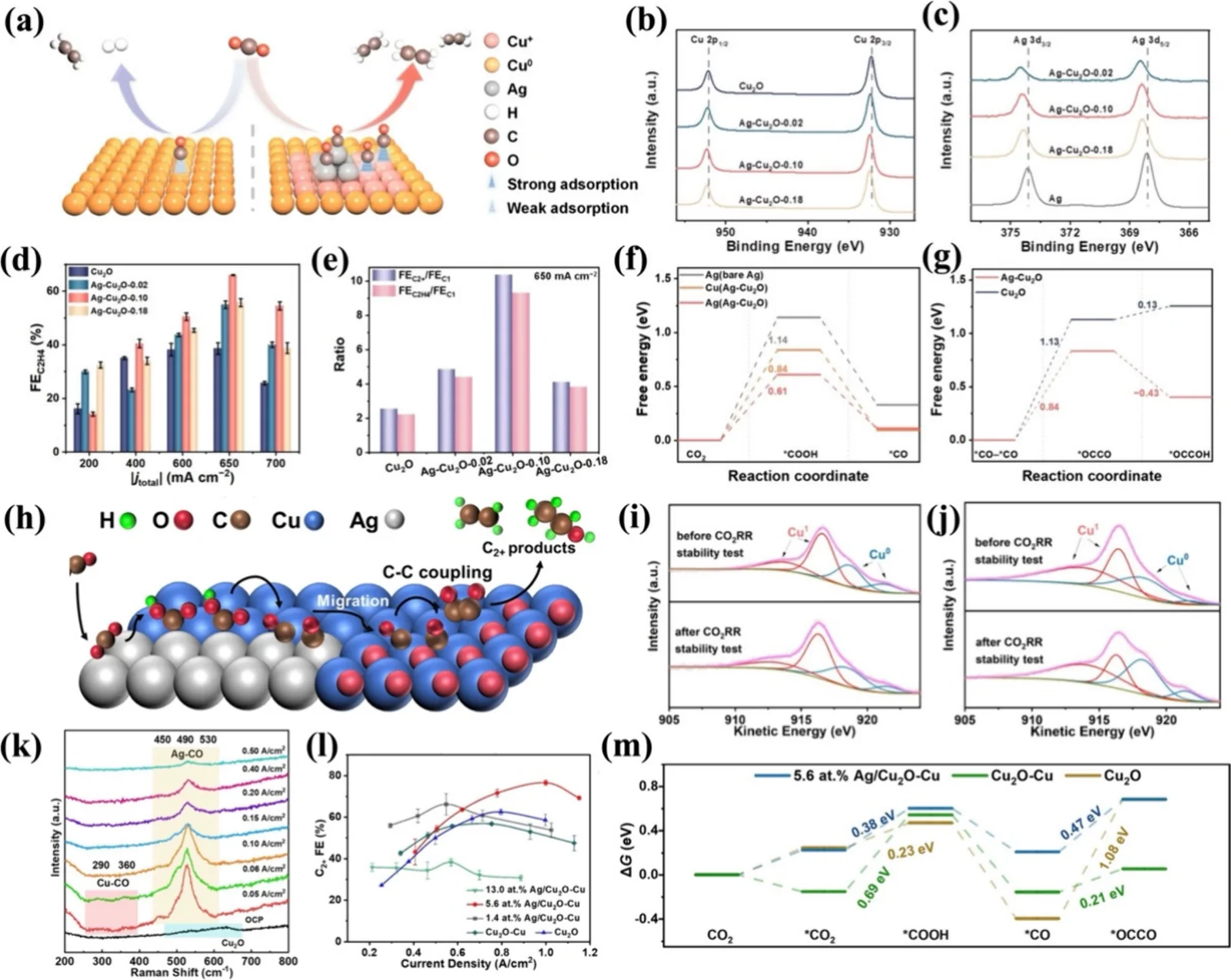
(a) Schematic illustration of product selectivity regulation on Cu_2_O and Ag–Cu_2_O interfacial catalysts. (b) Cu 2p XPS spectra of Cu_2_O and Ag–Cu_2_O catalysts with different Ag contents. (c) Ag 3d XPS spectra of Ag–Cu_2_O catalysts with different Ag contents and metallic Ag reference. (d) Faradaic efficiency of ethylene on Cu_2_O and Ag–Cu_2_O catalysts at different total current densities. (e) FE­(C_2+_)/FE­(C_1_) and FE­(C_2_H_4_)/FE­(C_1_) ratios of Cu_2_O and Ag–Cu_2_O catalysts at 650 mA cm^–2^. (f) DFT-calculated free-energy profiles for CO_2_-to-*CO conversion on Ag, Cu, and Ag–Cu_2_O models. (g) DFT-calculated free-energy profiles for *CO–*CO coupling and *OCCOH formation on Cu_2_O and Ag–Cu_2_O models. Panels (a–g) reproduced with permission from Ref 89. Copyright 2024 Wiley-VCH GmbH & Co. KGaA. (h) Schematic illustration of CO intermediate migration and C–C coupling on Ag/Cu_2_O–Cu heterointerfaces. (i, j) Cu LMM Auger spectra of Cu_2_O–Cu and Ag/Cu_2_O–Cu before and after CO_2_RR stability tests. (k) In situ Raman spectra of Ag/Cu_2_O–Cu collected at different current densities. (l) C_2+_ Faradaic efficiency of Cu_2_O, Cu_2_O–Cu, and Ag/Cu_2_O–Cu catalysts at different current densities. (m) DFT-calculated free-energy diagrams for CO_2_ activation, *COOH formation, *CO formation, and *OCCO formation on Cu_2_O, Cu_2_O–Cu, and Ag/Cu_2_O–Cu models. Panels (h–m) adapted with permission from Ref 90. Copyright 2025 American Chemical Society.

In terms of catalytic performance, the optimized Ag–Cu_2_O-0.10 catalyst achieved a C_2+_ Faradaic efficiency of 73.6% at 650 mA cm^–2^ in 3 M KCl neutral electrolyte, with an ethylene Faradaic efficiency of 66.0% and an ethylene partial current density of 429.1 mA cm^–2^ (Figure 5d). Compared with pristine Cu_2_O, the formation of the Ag/Cu_2_O interface significantly increased the C_2_H_4_/C_1_ and C_2+_/C_1_ ratios, indicating that Ag incorporation not only enhanced CO supply but also improved the efficiency of directing CO intermediates toward C–C coupling (Figure 5e). Furthermore, the DFT calculations shown in Figure 5f, g reveal that the Ag/Cu_2_O interface lowers the energy barriers for CO_2_ conversion to *COOH and subsequent *CO formation, while also facilitating *CO–*CO coupling to form *OCCO. These results suggest that the Ag/Cu_2_O interface actively participates in both CO generation and C–C coupling, rather than merely serving as a CO supplier in the tandem system.

Jiang et al. further demonstrated the role of Ag in stabilizing the Cu^+^/Cu^0^ interface.^90^ The Cu^+^/Cu^0^ interface is generally considered favorable for *CO adsorption and dimerization;^91–94^ however, under high-current-density CO_2_RR conditions, Cu^+^ species are readily reduced to Cu^0^, leading to gradual degradation of the interfacial structure and active sites. To address this challenge, Ag was introduced to construct a 5.6 atom % Ag/Cu_2_O–Cu catalyst containing abundant and stable Ag/Cu^+^/Cu^0^ heterointerfaces. In this structure, Ag not only promotes *CO intermediate generation and local *CO coverage but also stabilizes the Cu_2_O/Cu active interface, allowing the Cu^+^/Cu^0^ structure to be better preserved under high-current-density reaction conditions, as illustrated in Figure 5h.

Cu LMM Auger spectra collected before and after the stability test (Figure 5i,j) showed that Ag incorporation helped maintain the Cu^+^/Cu^0^ composition and suppressed excessive reduction of the Cu_2_O/Cu active interface during CO_2_RR. In situ Raman spectra (Figure 5k) further revealed more pronounced Ag–CO-related signals during the reaction, indicating that Ag incorporation increased *CO intermediate generation and surface coverage. The optimized catalyst achieved a C_2+_ Faradaic efficiency of 76.5 ± 1.2% at an ampere-level current density of 1.0 A cm^–2^ in a 1 M KOH flow cell (Figure 5l). The DFT results in Figure 5m further confirmed that the stable Ag/Cu^+^/Cu^0^ interface modulates the free energies of key intermediates, including *COOH, *CO, and *OCCO, thereby lowering the energy barriers associated with *CO formation and C–C coupling and ultimately promoting C_2+_ product formation.

Taken together, these two studies demonstrate that Ag incorporation in Cu-based catalysts plays a role that extends well beyond conventional CO supply in tandem catalysis. Ag–Cu bimetallic interfaces provide three key effects. First, Ag sites enhance CO/*CO generation and increase local *CO coverage. Second, electronic interactions between Ag and Cu_2_O or Cu^+^/Cu^0^ interfaces modulate the electronic structure of Cu active sites, thereby optimizing *CO adsorption and C–C coupling energetics. Third, Ag incorporation stabilizes Cu^+^/Cu^0^ active interfaces, improving catalytic stability under high-current-density operation.

### 4.2 Ag–Cu Single-Atom Alloy Catalysts: Promoting Intermediate Migration while Preserving Cu Active Sites

Compared with Ag/Cu bimetallic catalysts, Ag–Cu single-atom alloys (AgCu-SAAs) provide a more refined strategy for tandem catalyst design. In typical Ag nanoparticle-modified Cu catalysts, Ag can effectively promote CO_2_-to-CO conversion and increase the local CO concentration. However, excessive or aggregated Ag nanoparticles may partially cover the Cu surface, thereby reducing the number of Cu active sites available for C–C coupling. In addition, CO generated on Ag sites often needs to desorb into the electrolyte and subsequently readsorb onto neighboring Cu sites. This out-migration pathway can lead to CO loss, decreasing CO utilization efficiency and C_2+_ selectivity. Therefore, dispersing Ag as isolated atoms within a Cu matrix can improve Ag atom utilization, minimize blockage of Cu active sites, and establish a shorter and more direct CO/*CO intermediate-transfer pathway.

Du et al. proposed a cascade electrocatalyst combining AgCu single-atom alloy sites with Ag nanoparticles, denoted as AgCu SANP.^95^ In this design, Ag nanoparticles primarily function as CO-generating sites by reducing CO_2_ to CO, whereas AgCu-SAA sites regulate neighboring Cu sites for C–C coupling by tuning *CO adsorption through single-atom Ag doping. The key concept of this design is not simply to increase Ag loading, but to couple the CO-generation ability of Ag nanoparticles with the electronic and geometric regulation of Cu sites by AgCu-SAA. This enables CO generation and C–C coupling enhancement to occur synergistically within the same catalytic system, as illustrated in Figure 6a.

**Figure 6:**
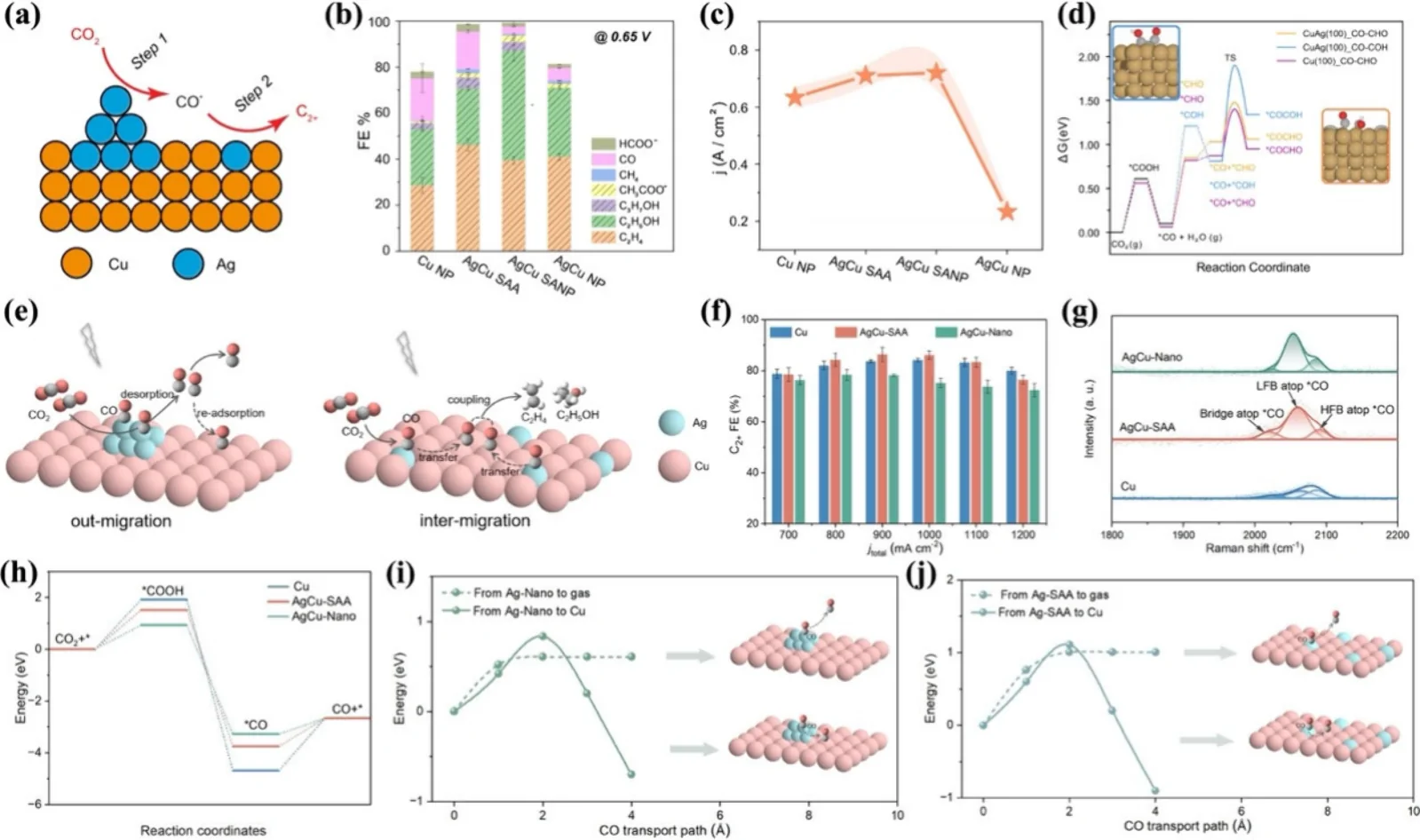
(a) Schematic illustration of cascade CO_2_RR on AgCu single-atom alloy/nanoparticle tandem catalysts. (b) Product Faradaic efficiency distributions of Cu nanoparticles, AgCu-SAA, AgCu-SANP, and AgCu nanoparticles at 0.65 V. (c) Current density comparison of Cu nanoparticles, AgCu-SAA, AgCu-SANP, and AgCu nanoparticles. (d) DFT-calculated reaction energy profiles for CO_2_-to-CO conversion and C–C coupling on Cu and AgCu models. Panels (a–d) reproduced with permission from Ref 95. under the terms of the Creative Commons Attribution 4.0 International (CC BY 4.0) License. Copyright 2023 The Author(s). (e) Schematic comparison of out-migration and inter-migration pathways for CO intermediates on AgCu-Nano and AgCu-SAA catalysts. (f) C_2+_ Faradaic efficiency of Cu, AgCu-SAA, and AgCu-Nano catalysts at different total current densities. (g) In situ Raman spectra in the CO stretching region showing atop *CO and bridge-bound *CO features on Cu, AgCu-SAA, and AgCu-Nano. (h) DFT-calculated free-energy profiles for CO_2_-to-*CO conversion on Cu, AgCu-SAA, and AgCu-Nano models. (i) DFT-calculated CO transport energy profiles from Ag-Nano sites to gas phase or Cu sites. (j) DFT-calculated CO transport energy profiles from Ag-SAA sites to gas phase or Cu sites. Panels (e–j) reproduced with permission from Ref 96. Copyright 2025 American Chemical Society.

In CO_2_RR flow-cell tests (Figure 6b,c), AgCu SANP achieved a C_2+_ Faradaic efficiency of 94 ± 4% at an operating current density of approximately 720 mA cm^–2^, significantly outperforming Cu nanoparticles, AgCu-SAA, and AgCu nanoparticles. Notably, although AgCu nanoparticles provide more Ag sites and enhance CO generation, their C_2+_ production decreased instead. This result indicates that excessive Ag can block or dilute Cu active sites, leaving an insufficient number of Cu sites available for C–C coupling. Therefore, the high C_2+_ selectivity of AgCu SANP originates from an optimized balance between Ag nanoparticles and AgCu-SAA sites: Ag nanoparticles supply local CO, while AgCu-SAA preserves and activates Cu-based C–C coupling sites, avoiding the Cu site loss caused by simply increasing Ag loading.

From the perspective of structural and electronic effects, incorporating isolated Ag atoms into Cu nanoparticles can introduce compressive strain into neighboring Cu–Cu bonds because of the larger atomic radius of Ag, thereby forming asymmetric Cu active sites. The DFT calculations shown in Figure 6d indicate that this asymmetric Ag–Cu coordination optimizes *CO adsorption on Cu sites and lowers the subsequent C–C coupling barrier. Therefore, the function of AgCu-SAA is not limited to increasing CO supply; it can also intrinsically enhance the ability of Cu sites to undergo *CO dimerization and C–C coupling. This explains why AgCu SANP outperforms Cu nanoparticles, AgCu-SAA-only catalysts, and AgCu nanoparticles.

Wang et al. further clarified the advantage of AgCu-SAA from the perspective of the CO intermediate migration pathway.^96^ In conventional Ag nanoparticle-modified Cu catalysts, CO generated on Ag sites tends to desorb first into the electrolyte because of weak CO–Ag binding, and then readsorb onto neighboring Cu sites, forming an indirect out-migration pathway. This transfer process can cause CO intermediate loss, increase CO Faradaic efficiency, and decrease C_2_^+^ selectivity. By contrast, in AgCu-SAA, Ag atoms are embedded or atomically dispersed within the Cu catalyst. Because no distinct blocking interface exists between Ag and adjacent Cu sites, CO generated on Ag sites can be directly transferred to neighboring Cu sites, forming a more efficient inter-migration pathway, as shown in Figure 6e.

In terms of catalytic performance (Figure 6f), AgCu-SAA achieved a C_2+_ Faradaic efficiency of 83.4% at 900 mA cm^–2^ and maintained 74.8% C_2+_ Faradaic efficiency even at a high current density of 1100 mA cm^–2^. In comparison, Ag nanoparticle-modified Cu increased CO generation but only moderately improved C_2_^+^ selectivity, while also exhibiting a higher CO Faradaic efficiency. This indicates that Ag nanoparticles mainly promote CO formation but do not efficiently direct CO toward C–C coupling on Cu. In situ Raman spectra (Figure 6g) showed that AgCu-SAA exhibits more pronounced Cu–CO, bridge-bound *CO, and low-frequency atop *CO signals than AgCu-Nano, indicating higher *CO coverage near Cu active sites. The DFT calculations shown in Figure 6h–j further demonstrate that, although AgCu-Nano is theoretically more favorable for CO/*CO generation, the inter-migration pathway from Ag sites to adjacent Cu sites is more favorable in AgCu-SAA. In contrast, in AgCu-Nano, CO more readily desorbs into the gas phase or electrolyte, leading to reduced intermediate utilization efficiency. This mechanistic distinction explains the superior CO utilization and C–C coupling capability of AgCu-SAA.

Overall, Ag–Cu tandem catalyst design has evolved from spatially separated CO-supply strategies toward increasingly integrated interfacial and atomic-scale cooperative regulation. Importantly, Ag–Cu bimetallic heterojunctions and AgCu single-atom alloys (SAAs) promote tandem CO_2_RR through mechanistically distinct modes of cooperation. In bimetallic heterojunctions, Ag and Cu/CuO_x_ remain as distinct phases connected through a finite interface. Their catalytic enhancement therefore relies primarily on interfacial charge redistribution, stabilization of favorable Cu oxidation states, modification of intermediate adsorption energetics, and the transfer or spillover of Ag-derived CO/*CO across the Ag–Cu phase boundary. Accordingly, the density, electronic structure, and structural stability of the heterointerface are critical factors governing intermediate utilization and overall tandem performance.

In contrast, AgCu SAAs contain isolated Ag atoms atomically dispersed within the Cu matrix rather than forming an extended Ag domains or Ag/Cu phase boundaries. Such atomic dispersion minimizes the geometric blockage and dilution of contiguous Cu ensembles required for C–C coupling, while locally perturbing the electronic structure and coordination environment of neighboring Cu atoms. The resulting asymmetric Ag–Cu coordination and associated lattice distortion can further modulate *CO adsorption and C–C coupling energetics. More importantly, the direct atomic scale proximity between isolated Ag and neighboring Cu sites enables short-range inter-migration of CO/*CO intermediates, reducing the need for the longer-range desorption, diffusion, and readsorption processes characteristic of spatially separated Ag/Cu components. Thus, whereas bimetallic heterojunctions primarily exploit phase-boundary-mediated electronic interactions and interfacial stabilization, and intermediate spillover, AgCu SAAs emphasize atomic-scale site proximity, preservation of Cu ensembles, local electronic regulation, and efficient intermediate transfer.

The Ag/Cu_2_O and Ag/Cu_2_O–Cu heterointerfaces discussed in Section 4.1 exemplify the former mechanism, in which Ag promotes C_2+_ formation through interfacial electronic modulation, increased *CO availability, and stabilization of catalytically favorable Cu^+^/Cu^0^ active interfaces. By contrast, the AgCu-SAA systems discussed in Section 4.2 illustrate how isolated Ag atoms can simultaneously regulate the properties of neighboring Cu sites and establish short intermediate-transfer pathways without excessively occupying the Cu ensembles required for C–C coupling. Collectively, these studies demonstrate that efficient Ag–Cu tandem catalysis involves more than simply maximizing CO generation. Rather, catalytic performance depends on matching the structural scale and mode of Ag–Cu cooperation with the required catalytic functions, including CO formation, *CO adsorption and migration, preservation of Cu active ensembles, interfacial or atomic-scale structural stability, and subsequent C–C coupling kinetics.

## 5 SAC–Cu Tandem Catalysts

In addition to Ag–Cu bimetallic interfaces and single-atom alloy strategies, SAC–Cu tandem systems have emerged as an attractive approach for promoting CO_2_RR toward C_2+_ products. Compared with metallic CO-generating components such as Ag and Au, single-atom catalysts or molecular M–N_4_ sites offer well-defined coordination environments, high atom utilization efficiency, and tunable CO_2_-to-CO activity through regulation of the central metal, ligand structure, and support interaction.^97–100^

However, high CO selectivity alone does not necessarily ensure efficient tandem catalysis. The CO-generation potential window must overlap with the potential range required for C–C coupling on Cu, and the generated CO/*CO intermediates must be efficiently transferred to nearby Cu sites before desorption or diffusion into the electrolyte. Therefore, efficient SAC–Cu tandem design requires the integrated regulation of CO generation, potential alignment, interfacial coupling, and *CO spillover pathways.

### 5.1 Potential Alignment and Interfacial Coupling in SAC–Cu Tandem Catalysts

Liu et al. first emphasized that operating potential alignment between CO-generating sites and Cu-based C–C coupling sites is a critical but often overlooked factor in tandem catalysts (Figure 7a).^101^ In general, Ag nanoparticles and certain molecular catalysts exhibit optimal CO generation activity at relatively mild cathodic potentials. However, *CO dimerization and C_2_H_4_ formation on Cu typically require more negative potentials. When Cu operates in the potential region most favorable for C–C coupling, these CO-generating components may instead favor the hydrogen evolution reaction, thereby failing to continuously supply CO and consequently reducing tandem efficiency.

**Figure 7:**
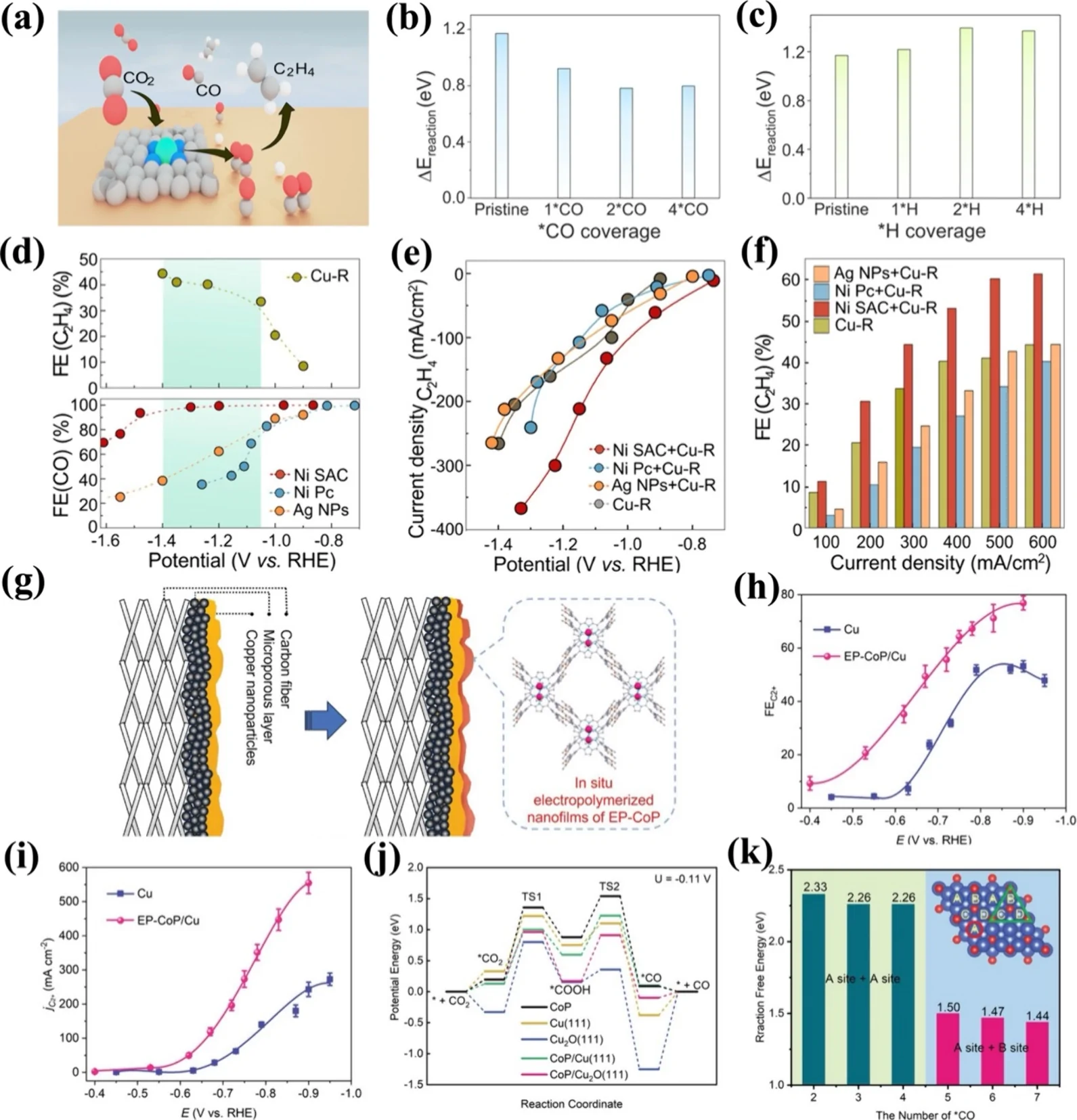
(a) Schematic illustration of Ni SAC + Cu tandem catalysts for CO_2_RR to ethylene. (b) DFT-calculated reaction barriers for *OCCO formation on Cu(111) under different *CO coverages. (c) DFT-calculated reaction barriers for *OCCO formation on Cu(111) under different *H coverages. (d) Potential-dependent FE­(C_2_H_4_) on Cu and FE­(CO) on Ni SAC, NiPc, and Ag nanoparticle CO-generating components. (e) Ethylene current density–potential curves of Ni SAC + Cu, NiPc + Cu, Ag nanoparticles + Cu, and Cu catalysts. (f) Ethylene Faradaic efficiency of Ni SAC + Cu, NiPc + Cu, Ag nanoparticles + Cu, and Cu catalysts at different current densities. Panels (a–f) reproduced from Ref 101. under the terms of the Creative Commons Attribution 4.0 International (CC BY 4.0) License. Copyright 2024 The Author(s). (g) Schematic illustration of in situ electropolymerization of cobalt porphyrin on Cu gas-diffusion electrodes to form EP-CoP/Cu tandem interfaces. (h) C_2+_ Faradaic efficiency of Cu and EP-CoP/Cu catalysts at different applied potentials. (i) C_2+_ partial current density of Cu and EP-CoP/Cu catalysts at different applied potentials. (j) DFT-calculated potential-energy profiles for CO_2_-to-CO conversion on CoP, Cu(111), Cu_2_O­(111), CoP/Cu(111), and CoP/Cu_2_O­(111) models. (k) DFT-calculated reaction free energies for C–C coupling under different local *CO coverages and adsorption configurations. Panels (g–k) adapted from Ref 102. under the terms of the Creative Commons Attribution 4.0 International (CC BY 4.0) License. Copyright 2024 The Author(s).

To clarify the importance of *CO coverage in C–C coupling, Liu et al. performed DFT calculations to analyze the activation barrier for *OCCO formation on Cu(111), as shown in Figure 7b. The results showed that increasing *CO coverage on the Cu surface significantly lowers the activation barrier for *CO–*CO coupling. In contrast, increased *H coverage raises the reaction barrier (Figure 7c), indicating that *H competes with *CO for adsorption sites and suppresses C–C coupling. These findings suggest that the key to efficient tandem catalysis is not merely increasing the overall reaction current, but rather maintaining high *CO coverage while suppressing HER within the potential window where Cu most effectively produces C_2_H_4_. Accordingly, an ideal CO-generating SAC should possess a broad CO generation potential window, allowing continuous CO supply even at more negative potentials.

Based on this concept, Liu et al. compared Ag nanoparticles, nickel phthalocyanine, and Ni SACs as CO-generating components. The results showed that Ni SACs possess a broader CO-generation potential range and better overlap with the C–C coupling potential window of Cu (Figure 7d). In flow-cell measurements (Figure 7e,f), the Ni SAC + Cu tandem catalyst achieved a C_2_H_4_ partial current density of approximately 370 mA cm^–2^ and a C_2_H_4_ Faradaic efficiency of approximately 62%. These results demonstrate that potential alignment determines whether the CO generated by SACs can be effectively supplied when Cu requires high *CO coverage, thereby affecting the overall tandem efficiency and C_2_H_4_ selectivity.

In addition to potential alignment, interfacial coupling between SACs and Cu, as well as *CO spillover, is another key factor governing the performance of SAC–Cu tandem catalysts. Wang et al. reported an EP-CoP/Cu tandem catalyst constructed by in situ electropolymerization, in which cobalt porphyrin monomers were directly electropolymerized onto a Cu gas-diffusion electrode to form a uniform electropolymerized cobalt porphyrin polymeric network (Figure 7g).^102^ Compared with conventional physically deposited molecular catalysts,^103,104^ in situ electropolymerization avoids molecular aggregation and burial of CoN_4_ active centers,^105^ while maintaining nanoscale contact between CoN_4_ sites and Cu nanocrystals. This intimate contact facilitates direct *CO intermediate spillover from Co sites to the Cu surface.

In the EP-CoP/Cu tandem system, CoN_4_ sites in EP-CoP function as CO-generating components, selectively reducing CO_2_ to CO or *CO intermediates. The adjacent Cu nanocrystals then serve as C–C coupling sites, converting transferred *CO into C_2+_ products. As shown in Figure 7h,i, this catalyst achieved a total current density of 726 mA cm^–2^ at −0.9 V versus RHE in a flow cell, with a C_2+_ Faradaic efficiency of 76.8% and an ethylene Faradaic efficiency of 43%. Compared with bare Cu, EP-CoP/Cu exhibited a higher C_2+_ partial current density and a positively shifted ethylene formation onset potential, indicating that the tightly coupled CoP/Cu tandem interface effectively enhances *CO supply and subsequent C–C coupling kinetics.

Theoretical calculations further revealed that the enhanced performance of EP-CoP/Cu arises from the combined effects of interfacial electronic structure and a *CO adsorption-energy gradient. The DFT results shown in Figure 7j,k indicate that the CoP/Cu_2_O­(111) interface provides more favorable reaction energetics for CO_2_-to-CO conversion. Interfacial electron transfer stabilizes the *COOH intermediate, thereby accelerating CO formation. Meanwhile, *CO adsorption is more favorable on Cu_2_O­(111) than on CoP, generating an adsorption-energy gradient from Co sites to the Cu surface. This gradient facilitates the transfer of *CO generated on CoN_4_ sites to neighboring Cu surfaces. As the local *CO coverage on Cu increases, the free-energy barrier for C–C coupling decreases, ultimately promoting ethylene and other C_2+_ product formation. This study demonstrates that, in molecular SAC–Cu tandem systems, high CO selectivity alone is insufficient; constructing a tightly coupled molecular–metal interface that promotes *CO spillover is also essential for improving CO utilization efficiency and C_2+_ selectivity.

### 5.2 Ligand-Engineered Molecular SACs: Regulating the Electronic Structure of Single-Atom Centers

Beyond interfacial coupling and operating-potential alignment, the electronic structure of the single-atom center itself is another critical factor governing the catalytic efficiency of SAC–Cu tandem systems. Recent studies have shown that, compared with conventional pyrolysis-derived M–N–C single-atom catalysts, molecular SACs with well-defined coordination structures enable more precise establishment of structure–activity relationships and therefore serve as important model systems for elucidating tandem CO_2_RR mechanisms.

Lu et al. employed thiophene-decorated nickel porphyrins to construct a series of molecular catalysts with well-defined Ni–N_4_ coordination structures, including NiTPP, NiTThP, and NiT_2_ThP. This study systematically investigated how sulfur-containing substituents influence the electronic structure of single-atom centers and tandem CO_2_RR activity, as shown in Figure 8a.^106^ Compared with conventional sulfur-doped M–N–C catalysts, in which the distribution and local structure of sulfur are difficult to identify,^107–109^ these molecular models preserve a fixed Ni–N_4_ coordination environment while varying only the number of surrounding thiophene ligands. This design enables direct investigation of ligand electronic effects on the single-atom center.

**Figure 8:**
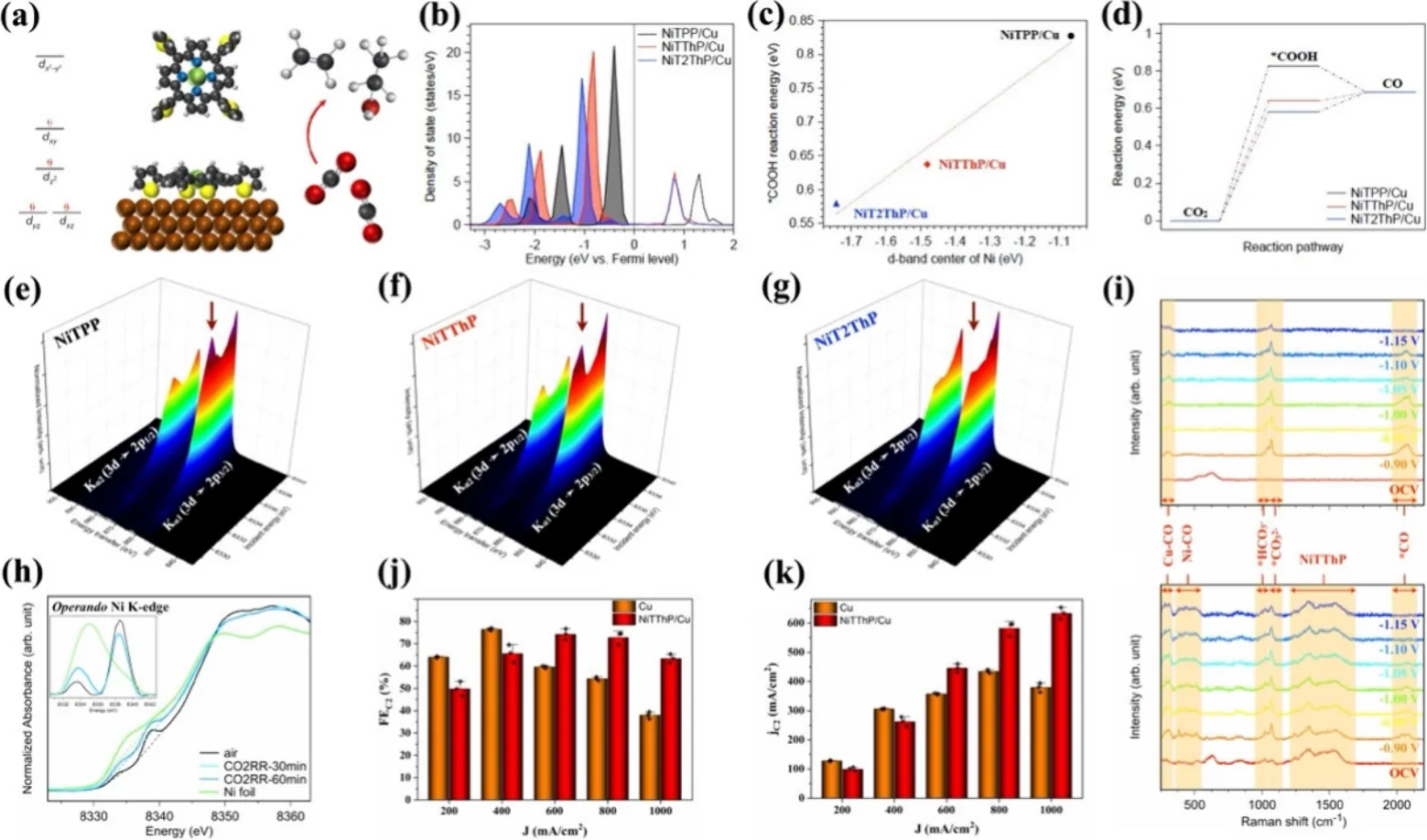
(a) DFT-optimized structure of thiophene-decorated Ni porphyrin on Cu(111) and schematic illustration of CO_2_RR intermediate formation. (b) Calculated density of states of NiTPP/Cu, NiTThP/Cu, and NiT_2_ThP/Cu. (c) Correlation between Ni d-band center and *COOH reaction energy for NiTPP/Cu, NiTThP/Cu, and NiT_2_ThP/Cu. (d) DFT-calculated reaction free-energy profiles for CO_2_-to-CO conversion on NiTPP/Cu, NiTThP/Cu, and NiT_2_ThP/Cu. (e–g) Resonant inelastic X-ray scattering maps of NiTPP, NiTThP, and NiT_2_ThP. (h) Operando Ni K-edge XANES spectra of NiTThP/Cu collected under CO_2_RR conditions. (i) Operando Raman spectra of Cu and NiTThP/Cu collected at different applied potentials. (j) C_2_ Faradaic efficiency of Cu and NiTThP/Cu at different current densities. (k) C_2_ partial current density of Cu and NiTThP/Cu at different current densities. Reproduced with permission from Ref 106. Copyright 2025 The Author(s).

The DFT results shown in Figure 8b–d reveal that thiophene substituents interact strongly with the Cu(111) surface, causing sulfur-containing ligands to preferentially approach the Cu surface and consequently modulate the electronic structure of the central Ni atom. As the number of thiophene substituents increases, the Ni 3d orbitals gradually shift downward, and the Ni d-band center decreases progressively from NiTPP to NiT_2_ThP. This trend exhibits a strong linear correlation with the *COOH formation energy. Since *COOH formation is a key step in CO_2_-to-CO conversion, lowering the Ni d-band center effectively reduces the *COOH formation barrier and enhances CO-generation activity. Reaction free-energy analysis further indicates that NiT_2_ThP/Cu exhibits the lowest barrier for *COOH formation while maintaining moderate CO desorption energy, which is beneficial for continuously supplying CO intermediates to neighboring Cu sites.

To verify this electronic-structure modulation mechanism, the authors further employed synchrotron-based Ni K-edge X-ray absorption near-edge structure (XANES), resonant inelastic X-ray scattering (RIXS), and extended X-ray absorption fine structure (EXAFS) analyses to examine the influence of thiophene ligands on the electronic state of the Ni center (Figure 8e–g). The results show that increasing the number of thiophene ligands does not simply generate high-valence Ni^3+^ species. Instead, thiophene substitution induces a pronounced ligand-hole character. This ligand-hole effect modified the Ni 3d orbital occupancy, shifts the Ni d-band center downward, and is consistent with the DFT results. These findings indicate that ligand engineering does not merely alter molecular geometry, but precisely regulates the electronic structure of the single-atom center through ligand electronic interactions, thereby improving CO_2_ activation capability.

Figure 8h further provides operando evidence for the stability and electronic evolution of the Ni molecular sites during CO_2_RR. Operando Ni K-edge XANES spectra collected under reaction conditions show that the absorption edge and spectral features of NiTThP/Cu remain close to those of the molecular Ni reference rather than shifting toward metallic Ni foil. This indicates that the Ni center largely preserves its isolated molecular coordination environment during CO_2_RR instead of being reduced into metallic Ni species. Therefore, the enhanced tandem activity can be attributed to ligand-regulated Ni–N_4_ molecular sites, rather than the formation of Ni nanoparticles or metallic Ni phases during operation. This result further supports the reliability of the molecular structure–activity relationship established using the thiophene-decorated Ni porphyrin model system.

In addition to theoretical and X-ray spectroscopic analyses, operando Raman spectroscopy in Figure 8i provides direct evidence for tandem reaction behavior. As the applied potential becomes more negative, signals associated with Ni–CO and Cu-bound *CO can be simultaneously observed. Among the investigated catalysts, NiTThP/Cu exhibits the most pronounced *CO-related signals, indicating that molecular Ni sites can continuously generate CO intermediates and effectively deliver them to neighboring Cu surfaces for subsequent C–C coupling, rather than allowing them to desorb directly into the electrolyte. This result confirms that ligand engineering not only enhances CO-generation efficiency but also improves CO intermediate utilization between SACs and Cu.

In flow-cell CO_2_RR tests (Figure 8j,k), NiTThP/Cu exhibited the best tandem catalytic performance, achieving a C_2_ Faradaic efficiency of approximately 74.3% and a C_2_ partial current density of approximately 445.8 mA cm^–2^ at high current density, both significantly higher than those of bare Cu. Notably, although NiT_2_ThP possesses the lowest *COOH formation energy, its overall C_2_ performance did not continue to improve. This observation indicates that maximizing CO-generation activity alone does not necessarily lead to optimal tandem performance. Instead, efficient tandem catalysis requires a balanced optimization of CO formation, CO transfer, and subsequent C–C coupling kinetics on Cu. Therefore, the best tandem performance originates from appropriate electronic-structure modulation rather than simply maximizing CO-generation activity.

Taken together, ligand engineering provides a design strategy distinct from conventional tuning of the central metal or support. By precisely regulating the electronic environment around single-atom centers, it becomes possible to establish a direct relationship among ligand structure, electronic structure, and catalytic activity. The thiophene-induced ligand-hole effect, Ni d-band modulation, and reduced *COOH formation energy collectively promote CO generation and subsequent tandem C–C coupling. This study not only establishes a structure–activity relationship for molecular SAC–Cu tandem systems, but also provides an important design principle for future SAC–Cu tandem catalysts with predictable electronic structures and high C_2+_ selectivity.

Overall, the development of SAC–Cu tandem catalysts has evolved from simply introducing CO-generating sites toward the integrated design of single-atom-center electronic structure, operating potential, interfacial distance, and intermediate transfer. These studies collectively indicate that efficient SAC–Cu tandem catalysis requires more than highly selective CO-generating sites. Instead, it relies on the integrated regulation of potential alignment, molecular–metal interfacial coupling, single-site coordination stability, and efficient *CO delivery to Cu for C–C coupling.

To provide a quantitative comparison of the strategy–performance relationships among different tandem architectures, the representative systems discussed in Sections 3–5 are summarized in Table 1. The comparison includes catalyst configuration, electrolyzer and electrolyte conditions, product Faradaic efficiency, current density, C_2_+/C_1_ product ratio, and operational stability. Because the reported performance metrics were obtained under different catalyst loadings, electrolytes, applied potentials or current densities, and stability protocols, the values should not be interpreted as a direct ranking of intrinsic catalytic activity. Instead, Table 1. highlights how different tandem construction strategies improve specific aspects of CO_2_RR performance.

**Table 1:** Comparison of Representative Cu-Based Tandem Catalyst Construction Strategies and Their CO_2_RR Performance toward C_2+_ Products under Flow-Cell Conditions (N.R., Not Reported)

Tandem strategy	Representative catalyst	Electrolyzer/electrolyte	Selectivity (FE)	Current density (mA cm^–2^)	C_2+_/C1 ratio	Stability	
Spatially separated/hierarchical CO management	Cu NWs/NC@Ag-PTFE	Flow cell/2.0 M KCl	(C_2+_) 87.5%	(Total) −519.0	10.42	≈ 50 h	ref 75
	Cu NWs@Ag-PTFE		(C_2_H_4_) 53.5%	(Total) −402.0	N.R.	N.R.	ref 75
			(C_2+_) 71.1%				
			(C_2_H_4_) 43.1%				
Spatially separated/wettability regulation	DDT–Ag/Cu	Flow cell/1 M KOH	(C_2+_) 74.09%	(C_2+_) −370.5	N.R.	≈ 13 h at 300 mA cm^–2^	ref 64
	Ag/Cu		(C_2+_) 76.02%	(C_2+_) −225.1	N.R.	≈ 6 h at 300 mA cm^–2^	ref 64
Spatially separated/facet-regulated tandem	layered Cu/Ag	Flow-cell/1 M KOH	(EtOH) 56.5%	(EtOH) −356.7	N.R.	≈ 5 h	ref 48
Bimetallic heterointerface	Ag–Cu_2_O-0.10	Flow cell/3 M KCl	(C_2+_) 73.6%	(C_2+_) −429.1	10.4	≈ 9 h at 600 mA cm^–2^	ref 89
			(C_2_H_4_) 66.0%				
Bimetallic heterointerface/Cu^+^/Cu^0^ stabilization	5.6 at.% Ag/Cu_2_O–Cu	Flow cell/1 M KOH	(C_2+_) 76.5%	(Total) −1000	N.R.	≈ 20 h at 500 mA cm^–2^	ref 90
SAA + NP cascade tandem	AgCu SANP	Flow cell/1 M KOH	(C_2+_) 94.4%	(C_2+_) −677	N.R.	13 h pulse electrolysis, total working time 9.75 h; FE(C_2_+) ∼98 → 88%	ref 95
Single-atom alloy tandem	AgCu-SAA	Flow cell/1 M KOH	(C_2+_) 83.4%	(C_2+_) −750.6	N.R.	≈ 17 h at 900 mA cm^–2^	ref 96
SAC–Cu/potential alignment	Ni SAC + Cu-R	Flow cell/1 M KHCO_3_	(C_2_H_4_) 62%	(C_2+_) −370	N.R.	≈ 14 h at 500 mA cm^–2^	ref 101
SAC–Cu/interfacial coupling	EP-CoP/Cu	Flow cell/1 M KOH	(C_2+_) 76.8%	(C_2+_) −533	N.R.	≈ 60 h at 200 mA cm^–2^ –0.68 V	ref 102
			(C_2_H_4_) 43%	(C_2_H_4_) −307		≈ 25 h at 430 mA cm^–2^ –0.75 V	
Ligand-engineered SAC–Cu	NiTThP/Cu	Flow cell/1 M KHCO_3_	(C_2+_) 74.3%	(C_2+_) −445.8	N.R.	≈ 20 h at 600 mA cm^–2^	ref 106

## 6 Conclusions and Perspectives

Cu-based electrocatalysts remain the most representative catalytic platform for CO_2_RR toward C_2+_ products because of their unique ability to retain *CO intermediates and promote C–C coupling. However, their practical performance is still limited by complex reaction pathways, insufficient *CO coverage under high-current-density operation, competing hydrogen evolution reaction (HER), and dynamic catalyst reconstruction, which collectively restrict C_2+_ selectivity, production rate, and operational stability. Tandem catalysis offers an effective strategy to address these limitations by integrating CO-generating components with Cu-based C–C coupling sites, thereby increasing local CO/*CO availability and promoting multicarbon product formation.

In this review, we summarized recent design strategies for Cu-based tandem catalysts, including spatially separated tandem architectures, Ag–Cu bimetallic interfaces and single-atom alloys, and SAC–Cu tandem systems. These studies demonstrate that efficient tandem CO_2_RR cannot be achieved simply by increasing CO generation. Instead, high-performance tandem catalysts require integrated regulation of CO transport, retention, adsorption, intermediate migration, interfacial electronic structure, operating-potential alignment, and C–C coupling pathways. These design principles have enabled remarkable improvements in C_2+_ selectivity and partial current density, especially under flow-cell conditions.

Despite substantial advances, Cu-based tandem CO_2_RR continues to face several fundamental challenges that must be addressed before its advantages can be translated into practical C_2_+ electrosynthesis. The first challenge concerns catalyst design and differs substantially among tandem architectures. In spatially separated systems, secondary catalytic components can efficiently convert CO_2_ to CO; however, overall tandem performance is determined not only by the rate of CO generation but also by the ability to retain, transport, and utilize the generated CO at neighboring Cu sites. In addition to minimizing CO loss over finite transport distances, morphological and microenvironmental engineering can prolong the residence time of CO within the catalyst layer and increase its probability of adsorption and further reduction on Cu. Improving the capture and utilization of tandem-generated CO therefore represents an important direction for future catalyst design. Meanwhile, secondary metal species or heteroatoms can modify the electronic structure of Cu and regulate *H adsorption, *CO binding, and subsequent C–C coupling.^110–113^ Integrating such Cu-site engineering with localized tandem CO generation may provide an effective strategy for directing CO toward C_2_+ formation rather than allowing its desorption or escape from the reaction interface.

For bimetallic tandem catalysts, the heterointerface itself constitutes an essential component of the active structure. Interfacial charge redistribution, stabilization of favorable Cu^+^/Cu^0^ states, and modulation of *CO adsorption energetics can directly influence intermediate utilization and C–C coupling. Importantly, such interfaces may dynamically evolve under strongly reducing CO_2_RR conditions. Reduction-induced phase reconstruction, metal migration, and changes in Cu oxidation state can continuously alter the density, composition, and chemical nature of interfacial active sites.^114,115^ Previous studies have further demonstrated that interfacial composition, atomic arrangement, and structural stability strongly influence intermediate adsorption and reaction selectivity.^116–118^ Future studies should therefore focus not only on constructing favorable heterointerfaces initially, but also on understanding and controlling their dynamic evolution under realistic operating conditions.

AgCu single-atom alloys (SAAs) present a distinct structural challenge because their atomic-scale cooperative effects depend on simultaneously maintaining isolated Ag sites and sufficiently large contiguous Cu ensembles for C–C coupling. Loss of atomic Ag dispersion, surface reconstruction, or excessive Ag incorporation may disrupt the optimized Ag–Cu coordination environment, decrease the availability of neighboring Cu ensembles, and diminish the short-range intermediate-transfer advantages of the SAA architecture.^119,120^ SAC–Cu tandem catalysts face analogous stability challenges associated with isolated coordination sites, such as M–N_4_ centers, whose reconstruction or aggregation during prolonged electrolysis may alter both their CO-generation activity and their cooperative interaction with Cu.^121,122^ Moreover, as Cu catalysts are increasingly optimized for high-rate C–C coupling across broader and more cathodic operating windows, matching the potential-dependent CO-generation activity of the tandem component with that of Cu becomes increasingly important. Future catalyst development should therefore move beyond maximizing the number or intrinsic activity of CO-generating sites and instead prioritize Cu active-site engineering, potential matching, dynamically stable interfaces, and persistent cooperative structures capable of sustaining efficient intermediate transfer.

The second major challenge concerns electrode and electrolyzer configuration, which should be regarded as an integral component of tandem-catalyst design rather than as an independent engineering consideration. In spatially separated and layered tandem electrodes, the sequence and thickness of the catalytic layers, catalyst loading, porosity, ionomer/binder distribution, and wettability collectively determine where CO is generated, how effectively it is retained, and whether it reaches Cu before desorbing or diffusing out of the catalyst layer. These factors become particularly important when tandem catalysts are translated from laboratory-scale electrodes to gas-diffusion electrodes, flow cells, and membrane-electrode assemblies. Furthermore, the electrolyte environment can fundamentally alter tandem mechanisms by regulating local pH, proton and water accessibility, *CO/*H competition, interfacial water structure, and Cu surface speciation. Consequently, an architecture optimized in alkaline or neutral flow cells may not retain the same tandem synergy under acidic conditions or in zero-gap MEA configurations.^123^ Future development should therefore integrate catalyst composition with electrode architecture, ionomer chemistry, electrolyte composition, membrane selection, and gas–liquid transport to preserve favorable intermediate-transfer pathways and interfacial microenvironments at industrially relevant current densities.

The third challenge is the translation of tandem catalysis from high-performing laboratory demonstrations to practical CO_2_ electrolysis. High C_2+_ Faradaic efficiency and partial current density must ultimately be achieved without excessive cell voltage or energy loss.^124,125^ Long-term stability also remains a major bottleneck because catalyst reconstruction, loss of active interfaces, aggregation of isolated metal sites, electrolyte flooding,^126,127^ salt precipitation,^128^ and gas-diffusion-layer degradation^129^ can progressively decrease catalytic activity and selectivity during high-current-density operation.^130,131^ Carbon utilization represents another critical system-level consideration,^132^ as carbonate formation, limited single-pass CO_2_ conversion, and the simultaneous production of gaseous and liquid C_2+_ products increase process complexity and downstream separation requirements.^133–136^ Thus, the material-level advantages of tandem catalysts should ultimately be evaluated together with their energy efficiency, carbon utilization, and structural durability under continuous operating conditions.

Overall, future advanced in Cu-based tandem CO_2_RR will require a transition from independently optimizing either the CO-generating component or the Cu catalyst toward the rational design of the entire cooperative catalytic interface and electrolyzer environment. Operando characterization under high-current-density conditions will be particularly important for tracking dynamic interface reconstruction, Cu oxidation-state evolution, single-atom stability, and CO/*CO migration between neighboring catalytic sites. In parallel, the evaluation of tandem systems should move beyond peak Faradaic efficiency alone and include tandem-relevant metrics such as C_2+_ partial current density, the C_2+_/CO ratio or CO-utilization efficiency, operating-potential window, single-pass carbon efficiency, cell voltage, and long-term preservation of the tandem interface. Establishing quantitative relationships among catalyst architecture, interface evolution, intermediate transport, electrolyte environment, and electrolyzer configuration will ultimately be essential for identifying which tandem strategies provide genuine advantages for scalable C_2+_ production.
